# Anti-Inflammatory and Cytotoxic Potential of New Phenanthrenoids from *Luzula sylvatica*

**DOI:** 10.3390/molecules25102372

**Published:** 2020-05-20

**Authors:** Maël Gainche, Isabelle Ripoche, François Senejoux, Juliette Cholet, Clémence Ogeron, Caroline Decombat, Ombeline Danton, Laetitia Delort, Marjolaine Vareille-Delarbre, Alexandre Berry, Marion Vermerie, Didier Fraisse, Catherine Felgines, Edwige Ranouille, Jean-Yves Berthon, Julien Priam, Etienne Saunier, Albert Tourrette, Yves Troin, Florence Caldefie-Chezet, Pierre Chalard

**Affiliations:** 1 Université Clermont-Auvergne, CNRS, SIGMA Clermont, ICCF, F-63000 Clermont-Ferrand, France; mael.gainche@sigma-clermont.fr (M.G.); yves.troin@sigma-clermont.fr (Y.T.); pierre.chalard@sigma-clermont.fr (P.C.); 2 Université Clermont-Auvergne, INRA, UNH, Unité de Nutrition Humaine, CRNH Auvergne, F-63000 Clermont-Ferrand, France; juliette.cholet@uca.fr (J.C.); clemence.ogeron@uca.fr (C.O.); caroline.decombat@uca.fr (C.D.); laetitia.delort@uca.fr (L.D.); marjolaine.vareille-delarbre@uca.fr (M.V.-D.); alexandre.berry@uca.fr (A.B.); marion.vermerie@uca.fr (M.V.); didier.fraisse@uca.fr (D.F.); catherine.felgines@uca.fr (C.F.); florence.caldefie-chezet@uca.fr (F.C.-C.); 3 Pharmaceutical Biology, Pharmacenter, University of Basel, Klingelbergstrasse 50, 4056 Basel, Switzerland; ombeline.danton@unibas.ch; 4 Greentech, Biopôle Clermont-Limagne, 63360 Saint-Beauzire, France; developpement@greentech.fr (E.R.); jeanyvesberthon@greentech.fr (J.-Y.B.); 5 Dômes Pharma, 3 Rue André Citroën, 63430 Pont-du-Château, France; j.priam@domespharma.com (J.P.); e.saunier@domespharma.com (E.S.); 6 AltoPhyto, 7 rue des Gargailles, 63370 Lempdes, France; albert.a.tourrette@gmail.com

**Keywords:** phenanthrene derivatives, antiproliferative activities, *Luzula sylvatica*

## Abstract

Phenanthrenoids have been widely described, in the Juncaceae family, for their biological properties such as antitumor, anxiolytic, anti-microbial, spasmolytic, and anti-inflammatory activities. The Juncaceae family is known to contain a large variety of phenanthrenoids possessing especially anti-inflammatory and cytotoxic properties. *Luzula sylvatica*, a Juncaceae species, is widely present in the Auvergne region of France, but has never been studied neither for its phytochemical profile nor for its biological properties. We investigated the phytochemical profile and evaluated the potential anti-inflammatory activities of *L. sylvatica* aerial parts extracts. A bioassay-guided fractionation was carried out to identify the most active fractions. Nine compounds were isolated, one coumarin **1** and eight phenanthrene derivatives (**2**–**9**), including four new compounds (**4**, **5**, **8** and **9**), from *n*-hexane and CH_2_Cl_2_, fractions. Their structures were established by HRESIMS, 1D and 2D NMR experiments. The biological properties, especially the anti-inflammatory/antioxidant activities (ROS production) and antiproliferative activity on THP-1, a monocytic leukemia cell line, of each compound, were evaluated. Three phenanthrene derivatives **4**, **6,** and **7** showed very promising antiproliferative activities.

## 1. Introduction

Juncaceae represents a large family of plants with nearly 500 different species distributed in seven genera, the most representative ones are the *Juncus* and the *Luzula* genera (around 350 and 110 species respectively).

Plants belonging to this family are widespread all around the world, the *Juncus* and *Luzula* genera grow in both hemispheres, generally in badly drained soils. Several studies described the use of Juncaceae species for the traditional treatment of dysuria, fidgetiness, irritability, insomnia, and inflammation [1]. In China, *J. inflexus* is commonly used for its sedative effect, as *J. effusus* and *J. conglomeratus* are used for skin diseases [2,3] in the Basque region. Considering *Luzula* species, no ethnopharmacological uses have been reported yet. The biological activities of Juncaceae seem mostly due to the presence of flavonoids [4], coumarins [5], terpenoids [6], and phenolic acid derivatives [7]. In addition, phenanthrene derivatives exerting potent anti-inflammatory [8,9] and cytotoxic activities [10,11] have also been identified in several members of the family. Such constituents are scarcely distributed in the plant kingdom and have mainly been reported in the Orchidaceae [11] and the Combretacae families [12,13]. Regarding Juncaceae, most of the chemical studies focused on the *Juncus* genus leading to the identification of more than 100 phenanthrenoids [1]. Even if a large variety of phenanthrenoids have been described, most of them possess a phenanthrene or a 9,10-dihydrophenanthrene backbone, often substituted at positions 2, 5 and 7 with hydroxyl, methyl, or vinyl groups. A vinyl group at position 5 on the phenanthrene skeleton could be considered as a chemotaxonomic marker for *Juncus* species [11]. 

To our knowledge, very limited investigations have been reported on the *Luzula* species especially on *Luzula sylvatica* (*L. sylvatica*) [10]. This species is widely present in the Auvergne area, a region of France which possesses diversified flora, particularly due to the variety of landscapes and climatic conditions. *L. sylvatica*, commonly named Great Wood-Rush, is a perennial plant, measuring between 30 and 110 cm, and growing mostly in wet soils (humid forest, in the shade). The numerous dark green basal leaves, possessing small white cils, are between 6 and 12 mm wide and 10–25 cm long. The inflorescence is loose, subdivided in open panicles, and flowers are brownish, sessile and group by 2–5 in glomerules [14]. 

Like most of the Juncaceae plants, *L. sylvatica* seems to contain phenanthrenoids [1,15]. This class of compounds is known to different have biological properties such as antitumor, anxiolytic, antimicrobial, spasmolytic and anti-inflammatory activities [11]. Considering the strong chemotaxonomical and biological interests of phenanthrenoids, the present study aimed to investigate the chemical composition of a methanolic extract from *L. sylvatica* aerial parts focusing particularly on this chemical class. In addition, anti-inflammatory and antiproliferative properties of the studied extract and its isolated compounds were evaluated.

## 2. Results and Discussion

**Figure 1:**
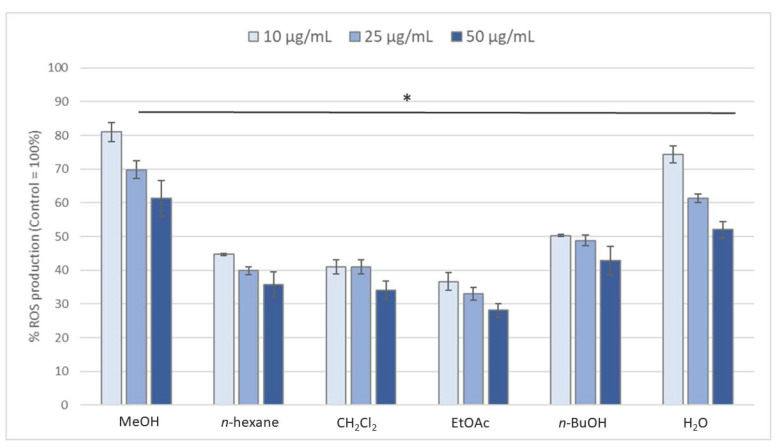
ROS production of blood leucocytes, incubated with the methanolic extract or fractions (10, 25 and 50 µg/mL) and stimulated with PMA (1 µM) for 1 h. Values are expressed as percentage of the control (cells incubated with PMA and without extract). Data are shown as means ± SEM (n = 3–6); * *p* < 0.05 compared with Control. All results are expressed as a percentage, with control (i.e., cells with PMA but without extract) normalized as 100%.

A methanolic extract, prepared from the aerial parts of *L. sylvatica,* was partitioned using increasing polarity liquid-liquid extraction. The five fractions ((*n*-hexane), dichloromethane (CH_2_Cl_2_), ethyl acetate (EtOAc), *n*-butanol (*n*-BuOH) and water) were tested for their anti-inflammatory/antioxidant activities by evaluating the production of ROS (reactive oxygen species) from blood leucocytes. All the fractions were able to decrease significantly the ROS production (Figure 1). The EtOAc, CH_2_Cl_2_ and *n*-Hex fractions were the most active ones (with a decrease of 60 to 75% compared to control). Analysis of the EtOAc fraction by LC-MS, LC-MS^2^, and UV spectrometry, in comparison with standards, led to the characterisation of luteolin, as its major constituent, as well as 1,3-*O*-dicaffeoylglycerol (ananasate) [16,17,18] and 3’,5,5’,7-tetrahydroxyflavanone [19]. Thus, the activity of EtOAc fraction could be due to the presence of luteolin, a flavone known for its anti-inflammatory properties [20,21]. 

**Figure 2:**
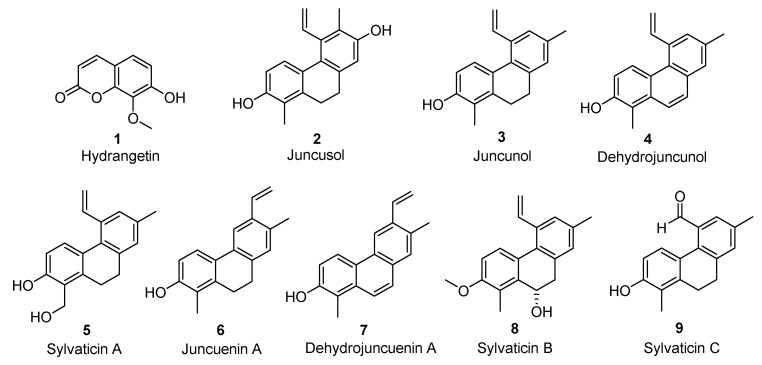
Isolated compounds from CH_2_Cl_2_ and *n*-hexane fractions.

Further purifications were performed on the *n*-hexane and the CH_2_Cl_2_ fractions which could potentially contain phenanthrene derivatives. From CH_2_Cl_2_ fraction, three compounds, namely juncusol (**2**) [1,22], compound **4** [23], and hydrangentin (**1**) [24,25], were isolated (Figure 2). 

*n*-hexane fraction was first purified by steric exclusion method using biobeads, then by column chromatography. Six major compounds were isolated, juncunol (**3**) [23], juncuenin A (**6**), dehydrojuncuenin A (**7**) [26] and three new compounds (**5**, **8**, and **9**), with original phenanthrene structures, were identified.

The HRESIMS of compound **4**, isolated as a pale-yellow oil, furnished deprotonated ion peak [M − H]^−^ at *m*/*z* 247.1127 (calcd 247.1128) corresponding to a molecular formula of C_18_H_16_O. The ^1^H NMR data showed the presence of three vinylic protons, six aromatic methine protons, two methyl groups and a hydroxyl group. A comparison of the NMR data of compound **4** with those obtained for juncunol and the HRESIMS data, suggested the presence of a phenanthrene backbone. The ^13^C NMR data confirm the presence of the vinylic function (*δ*_C_ 142.0 and 114.2 ppm) and the hydroxyl group (*δ*_C_ 151.1 ppm). HMBC correlations from H-13 (*δ*_H_ 5.78 and 5.44) to C-5 (*δ*_C_ 137.1) and from H-12 (*δ*_H_ 7.47) to C-6 (*δ*_C_ 130.6) suggested that the vinyl group was located at C-5. The HMBC correlations from H-6 (*δ*_H_ 7.45) and H-8 (*δ*_H_ 7.60) to C-14 (*δ*_C_ 21.3) suggested that the first methyl group was located at position 7. Moreover, a HMBC correlation from H-14 (*δ*_H_ 2.54) to C-7 (*δ*_C_ 134.9) confirmed this hypothesis. The HMBC correlation from H-8 (*δ*_H_ 7.60) to C-9 (*δ*_C_ 127.8) and the downshield chemical shifts of H-9 and H-10 protons (*δ*_H_ 7.69 and 7.89 ppm respectively) allowed us to confirm the skeleton of compound **4** as a phenanthrene backbone. The chemical shift of C-5a (*δ*_C_ 127.3) was determined based on the HMBC correlations from H-4 (*δ*_H_ 8.65), H-6 (*δ*_H_ 7.45), H-8 (*δ*_H_ 7.60) and H-9 (*δ*_H_ 7.69) to C-5a. The chemical shift of C-1a (*δ*_C_ 133.4) was confirmed by HMBC correlations from H-4 (*δ*_H_ 8.65) and H-9 (*δ*_H_ 7.69) to C-1a. The positions of the second methyl and the hydroxyl groups were determined by HMBC data. Correlations from H-11 (*δ*_H_ 2.61) to C-1 (*δ*_C_ 117.3), C-1a (*δ*_C_ 133.4) and C-2 (*δ*_C_ 151.1), from H-3 (*δ*_H_ 7.08) to C-1 (*δ*_C_ 117.3) and from H-4 (*δ*_H_ 8.65) to C-2 (*δ*_C_ 151.1) suggested that the second methyl group was located at C-1 and the hydroxyl group at C-2. The chemical shift of C-4a (*δ*_C_ 125.8) was confirmed by HMBC correlation from H-3 (*δ*_H_ 7.08) and H-10 (*δ*_H_ 7.89) to C-4a. The chemical shift of C-8a (*δ*_C_ 132.0) was confirmed by HMBC correlation from H-10 (*δ*_H_ 7.89) to C-8a. All these data confirmed the structure of compound **4** as 1,7-dimethyl-5-vinylphenanthrene-2-ol, named dehydrojuncunol.

Concerning compound **5,** the HRESIMS furnished deprotonated ion peak [M − H]^−^ at *m*/*z* 265.1242 corresponding to a molecular formula of C_18_H_18_O_2_ (calculated 265.1234). Comparison with the ^1^H and the ^13^C NMR spectra of compound **3** suggested that compound **5** possesses a 9,10-dihydrophenanthrene backbone with a vinyl group located at C-5, a methyl group at C-7 and a hydroxyl group at C-2. The ^1^H and ^13^C data established the presence of a hydroxylmethyl group (*δ*_H_ 5.01), singlet for two protons (*δ*_C_ 60.2). HMBC correlations from H-11 (*δ*_H_ 5.01) and C-2 (*δ*_C_ 155.3), C-1a (*δ*_C_ 138.4) and C-3 (*δ*_C_ 113.4) and the correlation from H-3 (*δ*_H_ 6.78) and C-2 (*δ*_C_ 155.3), C-1a (*δ*_C_ 138.4) suggested that the hydroxyl group is located at C-2 and the hydroxyl methylene group at C-1. The structure of the new isolated compound **5** was confirmed as 1-hydroxymethyl-7-methyl-5-vinyl-9,10-hydrophenanthrene-2-ol, named sylvaticin A.

For compound **8**, isolated as a pale-yellow oil, the molecular formula of C_19_H_20_O_2_ was established by HRESIMS (*m*/*z* 263.1426, [M + H − H_2_O]^+^; calculated *m*/*z* 263.1430) as well as the presence of a hydroxyl group. The chemical shift of a methine at *δ*_C_ 64.0 suggested the presence of the hydroxyl group on a non-aromatic carbon. ^1^H NMR data showed the presence of three vinylic protons, two methyl groups and a methoxy group. Comparison with the ^1^H and the ^13^C NMR spectra of compound **3** suggested that compound **8** is a 9,10-dihydrophenanthrene with a vinyl group located at C-5 and two methyl groups at C-1 and at C-7. The position of the methoxy group was determined by HMBC data. Correlations from H-4 (*δ*_H_ 7.56) and H-12 (*δ*_H_ 3.88) to C-2 (*δ*_C_ 157.1) suggested that the methoxy group is located at C-2. The structure of compound **8** was confirmed by NOESY. This experiment was performed in CD_3_OD in order to obtain two different chemical shifts for the protons of the two methyl groups (*δ*_H_ 2.33 and 2.35 respectively for H-11 and H-15) which had the same chemical shift in CDCl_3_ (*δ*_H_ 2.37). The data showed Overhauser effects between H-3 (*δ*_H_ 6.87) and H-12 (*δ*_H_ 3.88), H-4 (*δ*_H_ 7.56) and H-13 (*δ*_H_ 7.01), H-6 (*δ*_H_ 7.26) and H-14 (*δ*_H_ 5.72), H-8 (*δ*_H_ 7.09) and H-9 (*δ*_H_ 3.15), H-10 (*δ*_H_ 5.14) and H-11 (*δ*_H_ 2.37) as well as H-13 (*δ*_H_ 7.01) and H-14’(*δ*_H_ 5.28). The structure of the new compound **8** was determined as 2-methoxy-1,7-dimethyl-5-vinyl-9,10-dihydrophenanthrene-10-ol, named sylvaticin B. The CD spectrum of **8** showed a negative Cotton effect at 239 nm and a positive Cotton effect at 280 nm, in agreement with the (S) configuration of carbon 10 according to literature [27]. 

**Figure 3:**
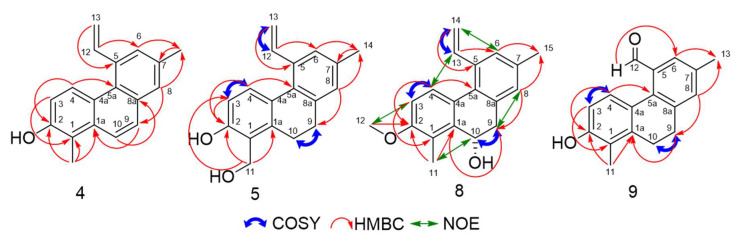
Key COSY, HMBC and NOESY correlations for compounds **4**, **5**, **8** and **9**.

The HRESIMS of compound **9** furnished a deprotonated ion peak [M − H]^−^ at *m*/*z* 251.1077 (calcd 251.1078) corresponding to a molecular formula of C_17_H_16_O_2_. As the ^1^H and^13^C NMR data were strongly similar to compound **3** NMR data, we could identify compound **9** as a 9,10-dihydrophenanthrene backbone possessing three substituents, two methyl groups at C-1 and C-7, and a hydroxyl group at C-2. The ^1^H and ^13^C NMR data showed the presence an aldehyde function with the corresponding carbon at *δ*_C_ 193.5 ppm (CH) and a singlet proton at *δ*_H_ 10.07 ppm. As previously reported in literature [28,29,30], the aldehyde function was located on C-5. This was confirmed by the HMBC correlations from H-12 (*δ*_H_ 10.07) to C-6 (*δ*_C_ 127.2). The position of all the substituents and the chemical shifts of all the protons and the carbons were attributed using HMBC and COSY correlations as described on Figure 3. The structure of the new compound **9** was determined as 2-hydroxy-1,7-dimethyl-9,10-dihydrophenanthrene-5-carbaldehyde, named sylvaticin C.

**Figure 4:**
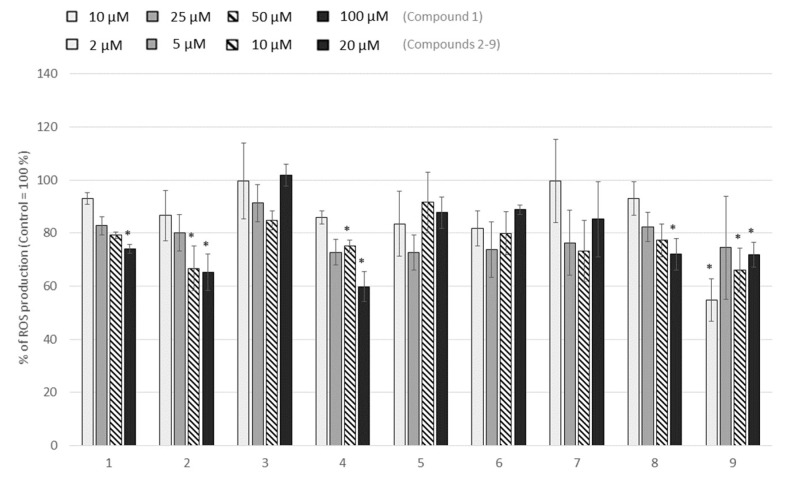
ROS production of blood leucocytes, incubated with the compounds (2, 5, 10 and 20 µM for compounds **2**–**9** and 10, 25, 50 and 100 µM for compound **1**) and stimulated with PMA (1 µM) for 1 h. Values are expressed as percentage of the control (cells incubated with PMA and without extract). Data are shown as means ± SEM (n = 3); * *p* < 0.05 compared with Control. All results are expressed as a percentage, with control (i.e., cells with PMA but without extract) normalized as 100%.

The nine isolated compounds were tested on leucocytes ROS production as described above for the five fractions. Four of them inhibited ROS production significantly in a dose-dependent manner: compounds **2**, **4**, **8**, and **1** (Figure 4), although the last one had to be tested at higher concentration to be effective.

In addition to their anti-inflammatory activity, phenanthrenes were known to exhibit promising in vitro antiproliferative activities on various cancer cell lines [9,31,32]. Thus, the cytotoxicity of the isolated compounds was evaluated with a resazurin assay on THP-1, a monocytic leukemia cell line.

Table 1 summarized the IC_50_ values obtained for each compound. With exception of compounds **1** and **9**, all components revealed a strong cytotoxic activity, with IC_50_ below 15 µM. The most effective compounds were **4**, **6,** and **7** (IC_50_ of 3, 6, and 5 µM, respectively).

## 3. Materials and Methods 

### 3.1. General Experimental Procedure

Nuclear magnetic resonance (NMR) spectra were recorded on an Avance III HD 400 MHz or 500 MHz (Bruker, Rheinstetten, Germany) spectrometer with CDCl_3_ as solvent (otherwise specified). All HPLC analyses were performed on an Agilent 1260 Infinity apparatus, with DAD detector (Agilent, Santa Clara, CA, USA) equipped with an Uptisphere C18-3 (250 × 4.6 mm, 5 µm) column (Interchim, Montluçon, France). LC-MS were carried out on an UHPLC Ultimate 3000 RSLC chain and an Orbitrap Q-Exactive (Thermo Scientific, Waltham, MA, USA) with the column mentioned above. Preparative chromatography was performed with a Varian prepstar Model SD-1 (Varian, Santa Clara, CA, USA) on an Uptisphere C18-3 (250 × 21.6 mm, 5 µm) semi-preparative column (Interchim). For all analyses, the mobile phase was a mixture of 0.1% (v:v) formic acid in water (phase A) and 0.1% (v:v) formic acid in acetonitrile (phase B). The gradient of phase A was 100% (0 min), 80% (10 min), 73% (35 min), 0% (40–50 min) and 100% (51–60 min). The flow rate was 0.8 mL/min, and the injection volume was 5 µL. 

Flash chromatography was performed on a SPOT II FLASH (Interchim) with a monochromator detector. All chemical standards and references were purchased from Extrasynthese (Genay, France). 

ECD spectra were recorded in methanol (200 μg/mL) on a Chirascan CD spectrometer using 1 mm path precision cells (110 QS, Hellma Analytics, Müllheim, Germany).

### 3.2. Plant Material

The aerial parts of *L. sylvatica* were collected in June 2017 (Cantal). A voucher specimen (CLF 110940) was deposited at the UniVegE herbarium of the Clermont Auvergne University.

### 3.3. Extraction and Isolation

Aerial parts of *L. sylvatica* (1.6 kg) were air-dried at room temperature in a dark room, powdered, then extracted with methanol (3 × 20 L each 24 h) and dried under *vacuo* to yield a greenish solid crude extract (195 g). This crude extract was then dissolved in water and extracted three times with increase solvent polarity to give five fractions: *n*-hexane (16.3%), CH_2_Cl_2_ (0.5%), EtOAc (2.0%) *n*-butanol (20.6%) and water (60.6%). 

A part of the *n*-hexane fraction (12.11 g out of 21.87 g) was then partitioned with Biobeads SX-3 column chromatography (from Bio-Rad, Hercules, USA) in THF to afford 4 fractions (1–4). Fraction 4 (216.3 mg) was then partitioned through a silica gel column chromatography with cyclohexane - EtOAc as eluent (from 95:5 to 0:100, v:v) to afford six fractions (1 to 6). Fraction 1 contained only one major product: juncunol (26.3 mg) (**3**). Fraction 2 (77.2 mg) was subjected to semi-preparative chromatography with MeCN-H_2_O + 0.1% formic acid (75:25, v:v) to obtain juncuenin A (**6**) (4.9 mg) and dehydrojuncuenin A (**7**) (2 mg). Fraction 3 (29.6 mg) was purified by semi-preparative chromatography using MeCN-H_2_O + 0.1% formic acid (75:25, v:v) as eluent to obtain sylvaticin B (**8**) (3.1 mg) and sylvaticin C (**9**) (1.7 mg). Fraction 5 (36.7 mg) was purified with a silica gel chromatography column with cyclohexane-EtOAc (75:25, v:v) as eluent to afford sylvaticin A (**5**) (20.8 mg).

Part of the CH_2_Cl_2_ fraction (631 mg out of 983 mg) was subjected to a flash chromatography (normal phase) to afford 11 fractions (1 to 11). Fraction 2 was purified by silica gel column chromatography with cyclohexane-EtOAc (8:2 then 6:4, v:v) to afford juncusol (**2**) (4 mg). Fraction 3 (34.7 mg) was purified by silica gel column chromatography with cyclohexane-EtOAc (from 8:2 to 5:5, v:v) to obtain dehydrojuncunol **4** (3.4 mg). Fraction 5 was purified by silica gel chromatography column with cyclohexane-EtOAc-MeOH (from 6:4:0 to 0:9:1, v:v) to obtain hydrangetin (**1**) (25.3 mg).

*1,7-Dimethyl-5-vinyl-phenanthrene-2-ol/ dehydrojuncunol* (**4**). Pale yellow oil; ^1^H and ^13^C NMR data, Table 2, HRMS *m*/*z* 247.1127 [M − H]^−^ (calcd for C_18_H_16_O, 247.1128), more data in Supplementary Materials.

*1-Hydroxymethyl-7-methyl-5-vinyl-9,10-hydrophenanthrene-2-ol/ sylvaticin A (***5***)*. Pale yellow oil; ^1^H and ^13^C NMR data, Table 2, HRMS *m*/*z* 265.1242 [M − H]^−^ (calcd for C_18_H_18_O_2_, 265.1234).

*2-Methoxy-1,7-dimethyl-5-vinyl-9,10-dihydrophenanthrene-10-ol/ sylvaticin B* (**8**). Pale yellow oil; ^1^H and ^13^C NMR data, Table 2, HRMS *m*/*z* 263.1426 [M + H − H_2_O]^+^ (calcd for C_19_H_18_O, 263.1430).

*6-Hydroxy-2,5-dimethyl-9,10-dihydrophenanthrene-4-carbaldehyde/ sylvaticin C* (**9**). Pale yellow oil; ^1^H and ^13^C NMR data, Table 2, HRMS *m*/*z* 251.1077 [M − H]^−^ (calcd for C_17_H_16_O_2_, 251.1078).

### 3.4. Blood Leucocytes ROS Production 

Blood was collected from healthy human volunteers (n = 3–6; Etablissement Français du Sang, EFS, Clermont-Ferrand, France). Donors gave their written informed consent for the use of blood samples for research purposes under EFS contract n°16-21-62 (in accordance with the following articles L1222-1, L1222-8, L1243-4 and R1243-61 of the French Public Health Code). Blood leukocytes were obtained by hemolytic shock using ammonium chloride solution. Leucocytes were then washed with RPMI 1640 medium (GIBCO, ThermoFisher Scientific), centrifuged and suspended in supplemented RPMI (fetal bovine serum (FBS) 10%, gentamicin 50 μg/mL and glutamine (Gln) 2 mM). Cells were placed in 96-well plates (Cell Wells™, Corning, NY, USA), incubated with the extracts or the compounds and dihydrorhodamine 123 (DHR 123, 1 μM, Cayman Chemical Company, Ann Arbor, MI), and stimulated, or not, by 1 µM phorbol myristate acetate (PMA) for 60 min. The fluorescence intensity of rhodamine 123, which is the product of dihydrorhodamine 123 oxidation by ROS, was recorded (excitation/emission: 485/538 nm) using a microplate fluorometric reader (Tecan Spark®, Mämmedorf, Switzerland) [33,34].

### 3.5. Cytotoxicity Evaluation of the Compounds 

The human monocytic leukemia cell line, THP-1 (American Type Culture Collection) was cultured and propagated at 37 °C in a humidified atmosphere of 5% CO_2_ in a RPMI 1640 medium supplemented with 10% FBS, gentamicin 50 μg/mL and Gln 2 mM. Cells were placed in 96-well plates and incubated with the compounds for 24 h. Then resazurin (25 µg/mL) was added to the medium to track their viability. Fluorescence (excitation/emission: 544/590 nm) was recorded for 2 h using a microplate fluorometric reader (Tecan Spark®) [35].

## 4. Conclusions

Phenanthrenoids are common secondary metabolites in plants of the Juncaceae family, possessing antiproliferative activities. From aerial parts of *L. sylvatica*, a Juncaceae species widely present in Auvergne region in France, we isolated nine compounds from the apolar *n*-hexane and CH_2_Cl_2_ fractions: coumarin **1** and eight phenanthrenoids (**2**–**9**). Among them, four new compounds were characterized: dehydrojuncunol **4**, sylvaticin A **5**, sylvaticin B **8**, uncommonly substituted at C-10, and sylvaticin C **9**. We demonstrated that phenanthrenoids **2**, **4,** and **8** inhibited ROS production significantly in a dose-dependent manner. 

Compounds **4**, **6**, and **7** showed moderate cytotoxic effects on THP-1 cells with IC_50_ lower than 6 µM. Further investigations will be carried out on the most active compounds, especially on new phenanthrenoid **4** in order to study its antiproliferative activity on other cancer cells and to explore its mechanism of action. 

## Figures and Tables

**Table 1:** Cytotoxic effect of compounds **1**–**9** in THP-1 cells reported as IC_50_ in µM. Cells metabolic activity was determined after 24 h of incubation with or without compounds at different concentrations (2, 5, 10 and 20 µM for compounds **2**–**9** and 10, 25, 50 and 100 µM for compound **1**) by resazurin assay (n = 3). Doxorubicine was used as a positive control at 1 µM for our experiments. At 1 µM, the viability of THP-1 decreased at around 80% (21.4 ± 3% of the fluorescence after 24 h of incubation, with negative control normalized as 100%).

Compound	IC_50_ (µM)
**1**	>100
**2**	10
**3**	13
**4**	3
**5**	11
**6**	6
**7**	5
**8**	10
**9**	>20

**Table 2:** ^1^H and ^13^C data for compounds **4**, **5**, **8** and **9** (in CDCl_3_).

	Compound 4		Compound 5		Compound 8		Compound 9	
Position	δ_C_ type	δ_H_ (*J* in Hz)	δ_C_ type	δ_H_ (*J* in Hz)	δ_C_ type	δ_H_ (*J* in Hz)	δ_C_ type	δ_H_ (*J* in Hz)
1	117.3, C		126.8, C		124.1, C		121.7, C	
2	151.1, C		155.3, C		157.1, C		154.5, C	
3	114.8, CH	7.08, d (9.1)	113.5, CH	6.78, d (8.5)	109.2, CH	6.87, d (8.4)	112.7, CH	6.76, d (8.4)
4	127.4 CH	8.65, d (9.1)	130.6, CH	7.49, d (8.5)	128.5, CH	7.56, d (8.4)	129.4, CH	6.92, d (8.4)
5	137.1 C		135.1, C		135.4 C		140.1, C	
6	130.6, CH	7.45, s	127.4, CH	7.25, s	128.3, CH	7.26, s	127.2, CH	7.64, s
7	134.9, C		136.0, C		136.7, C		136.3, C	
8	128.2, CH	7.60, s	127.6, CH	7.00, s	130.1, CH	7.09, s	133.1, CH	7.30, s
9	127.8, CH	7.69, d (9.1)	30.0, CH_2_	2.69, m	38.2, CH_2_	3.15, dd (16, 2.8)	29.1, CH_2_	2.78, m
9’						2.95, dd (16, 3)		
10	122.8, CH	7.89, d (9.1)	25.1, CH_2_	2.69, m	64.0, CH	5.14, m	25.5, CH_2_	2.82, m
11	11.3, CH_3_	2.61, s	60.2, CH_2_	5.01, s	11.2, CH_3_	2.37, s	11.8, CH_3_	2.30, s
12	142.0, CH	7.47, dd (17.3, 10.7)	138.8, CH	6.93, dd (17.4, 10.8)	55.8, CH_3_	3.88, s	193.6, CH	10.07, s
13	114.2, CH_2_	5.78, dd (17.3, 1.7)	114.1, CH_2_	5.70, dd (17.4, 1.3)	139.1, CH	7.01, dd (17, 10)	21.1, CH_3_	2.41, s
13’		5.44, dd (10.7, 1.7)		5.25 dd (10.8, 1.3)				
14	21.3, CH_3_	2.54, s	21.2, CH_3_	2.37, s	114.3, CH_2_	5.72, dd (17, 2)		
14’						5.28, dd (10, 2)		
15					21.1, CH_3_	2.37, s		
1a	133.4, C		138.4, C		138.2, C		139.5, C	
4a	125.8, C		121.6, C		125.4, C		124.4, C	
5a	127.3, C		131.1, C		129.9, C		136.7, C	
8a	132.0, C		138.5, C		132.9, C		133.0, C	
OH		4.94 brs						5.12, brs

## Supplementary Materials

The NMR data are available online.
