# Supplementary material for: Anti-Inflammatory and Cytotoxic Potential of New Phenanthrenoids from Luzula sylvatica
Source: Molecules. 2020 May 20;25(10):2372. doi: 10.3390/molecules25102372 (PMC7288028; doi:10.3390/molecules25102372)
Supplement: Supplementary file 1 [file molecules-25-02372-s001.pdf]

Supplementary materials

Anti-inflammatory and cytotoxic potential of new phenanthrenoids from *Luzula sylvatica*

Maël Gainche [1], Isabelle Ripoche [1,*], François Senejoux [2,*], Juliette Cholet [2], Clémence Ogeron [2], Caroline Decombat [2], Ombeline Danton [3], Laetitia Delort [2], Marjolaine Vareille-Delarbre [2], Alexandre Berry [2], Marion Vermerie [2], Didier Fraisse [2], Catherine Felgines [2], Edwige Ranouille [4], Jean-Yves Berthon [4], Julien Priam [5], Etienne Saunier [5], Albert Tourrette [6], Yves Troin [1],.Florence Caldefie-Chezet [2] and Pierre Chalard [1]

[1] Université Clermont-Auvergne, CNRS, SIGMA Clermont, ICCF, F-63000 Clermont-Ferrand, France; mael.gainche@sigma-clermont.fr (M.G.); yves.troin@sigma-clermont.fr (Y.T.), pierre.chalard@sigma-clermont.fr (P.C.)

[2] Université Clermont-Auvergne, INRA, UNH, Unité de Nutrition Humaine, CRNH Auvergne, F-63000 Clermont-Ferrand, France; juliette.cholet@uca.fr (J.C.); clemence.ogeron@uca.fr (C.O); caroline.decombat@uca.fr (C.D.); laetitia.delort@uca.fr (L.D.); marjolaine.vareille-delarbre@uca.fr (M.V.-D.); alexandre.berry@uca.fr (A.B.); marion.vermerie@uca.fr (M.V.); didier.fraisse@uca.fr (D.F.); catherine.felgines@uca.fr (C.F.); florence.caldefie-chezet@uca.fr (F. C.-C.)

[3] Pharmaceutical Biology, Pharmacenter, University of Basel, Klingelbergstrasse 50, 4056 Basel, Switzerland; ombeline.danton@unibas.ch (O.D.)

[4] Greentech, Biopôle Clermont-Limagne, 63360 Saint-Beauzire, France, developpement@greentech.fr (E.R.); jeanyvesberthon@greentech.fr (J.-Y.B.)

[5] Dômes Pharma, 3 Rue André Citroën, 63430 Pont-du-Château, France, j.priam@domespharma.com (J.P.); e.saunier@domespharma.com (E.S.)

[6] AltoPhyto, 7 rue des gargailles, 63370 Lempdes, France, albert.a.tourrette@gmail.com (A.T.)

* Correspondence: isabelle.ripoche@sigma-clermont.fr (I.R.); francois.senejoux@uca.fr (F.S.)

Figure S1. ^{1}H-NMR spectrum of hydrangetin (**1**) (500MHz, $CDCl_3$) 3
Figure S2. ^{13}C-NMR Spectrum of hydrangetin (**1**) (100MHz, $CDCl_3$) 3
Figure S3. HRESIMS of hydrangetin (**1**) (negative ionisation mode) 4
Figure S4. ^{1}H-NMR spectrum of juncusol (**2**) (400MHz, $CDCl_3$) 4
Figure S5. ^{13}C-NMR spectrum of juncusol (**2**) (100MHz, $CDCl_3$) 5
Figure S6. HRESIMS of juncusol (**2**) (negative ionisation mode) 5
Figure S7. ^{1}H-NMR spectrum of juncunol (**3**) (400MHz, $CDCl_3$) 6
Figure S8. ^{13}C-NMR spectrum of juncunol (**3**) (100MHz, $CDCl_3$) 6
Figure S9. HRESIMS of juncunol (**3**) (negative ionisation mode) 7
Figure S10. ^{1}H-NMR spectrum of 1,7-dimethyl-5-vinyl phenanthren-2-ol (**4**) (400MHz, $CDCl_3$) 7
Figure S11. ^{13}C-NMR spectrum of 1,7-dimethyl-5-vinyl phenanthren-2-ol (**4**) (100MHz, $CDCl_3$) 8
Figure S12. ^{1}H-^{13}C HSQC spectrum of 1,7-dimethyl-5-vinyl phenanthren-2-ol (**4**) (400MHz, $CDCl_3$) 8
Figure S13. ^{1}H-^{13}C HMBC spectrum of 1,7-dimethyl-5-vinyl phenanthren-2-ol (**4**) (400MHz, $CDCl_3$) 9
Figure S14. HRESIMS of 1,7-dimethyl-5-vinyl phenanthren-2-ol (**4**) (negative ionisation mode) 9
Figure S15. ^{1}H-NMR spectrum of 1-hydroxymethyl-7-methyl-5-vinyl-9,10-hydrophenanthren-2-ol (**5**) (400MHz, $CDCl_3$) 10
Figure S16. ^{13}C-NMR spectrum of 1-hydroxymethyl-7-methyl-5-vinyl-9,10-hydrophenanthren-2-ol (**5**) (100MHz, $CDCl_3$) 10
Figure S17. ^{1}H-^{1}H COSY spectrum of 1-hydroxymethyl-7-methyl-5-vinyl-9,10-hydrophenanthren-2-ol (**5**) (400MHz, $CDCl_3$) 11
Figure S18. ^{1}H-^{13}C HSQC spectrum of 1-hydroxymethyl-7-methyl-5-vinyl-9,10-hydrophenanthren-2-ol (**5**) (400MHz, $CDCl_3$) 11

Figure S19. ^{1}H-^{13}C HMBC spectrum of 1-hydroxymethyl-7-methyl-5-vinyl-9,10-hydrophenanthren-2-ol (**5**) (400MHz, $CDCl_3$) 12
Figure S20. HRESIMS of 1-hydroxymethyl-7-methyl-5-vinyl-9,10-hydrophenanthren-2-ol (**5**) (negative ionisation mode) 12
Figure S21. ^{1}H-NMR spectrum of juncuenin A (**6**) (400MHz, $CDCl_3$) 13
Figure S22. ^{13}C-NMR spectrum of juncuenin A (**6**) (100MHz, $CDCl_3$) 13
Figure S23. HRESIMS of juncuenin A (**6**) (negative ionisation mode) 14
Figure S24. ^{1}H-NMR spectrum of dehydrojucuenin A (**7**) (400MHz, $CDCl_3$) 14
Figure S25. ^{13}C-NMR spectrum of dehydojuncuenin A (**7**) (100MHz, $CDCl_3$) 15
Figure S26. ^{1}H-NMR spectrum of 2-methoxy-1,7-dimethyl-5-vinyl-9,10-dihydrophenanthren-10-ol (**8**) (400MHz, $CDCl_3$) 15
Figure S27. ^{13}C-NMR JMOD spectrum of 2-methoxy-1,7-dimethyl-5-vinyl-9,10-dihydrophenanthren-10-ol (**8**) (100MHz, $CDCl_3$, CH and CH_3 down, C and CH_2 up) 16
Figure S28. ^{1}H-^{1}H COSY spectrum of 2-methoxy-1,7-dimethyl-5-vinyl-9,10-dihydrophenanthren-10-ol (**8**) (400MHz, $CDCl_3$) 16
Figure S29. ^{1}H-^{13}C HSQC spectrum of 2-methoxy-1,7-dimethyl-5-vinyl-9,10-dihydrophenanthren-10-ol (**8**) (400MHz, $CDCl_3$) 17
Figure S30. ^{1}H-^{13}C HMBC spectrum of 2-methoxy-1,7-dimethyl-5-vinyl-9,10-dihydrophenanthren-10-ol (**8**) (400MHz, $CDCl_3$) 17
Figure S31. ^{1}H-NMR spectrum of 2-methoxy-1,7-dimethyl-5-vinyl-9,10-dihydrophenanthren-10-ol (**8**) (500MHz, CD_3OD) 18
Figure S32. ^{1}H-^{1}H NOESY spectrum of 2-methoxy-1,7-dimethyl-5-vinyl-9,10-dihydrophenanthren-10-ol (**8**) (400MHz, CD_3OD) 18
Figure S33. Experimental ECD spectra of compound (**8**) 19
Figure S34. HRESIMS of 2-methoxy-1,7-dimethyl-5-vinyl-9,10-dihydrophenanthren-10-ol (**8**) (positive ionisation mode) 24
Figure S35. ^{1}H-NMR spectrum of 2-hydroxy-1,7-dimethyl-9,10-dihydrophenanthrene-5-carbaldehyde (**9**) (400MHz, $CDCl_3$) 25
Figure S36. ^{13}C-NMR spectrum of 2-hydroxy-1,7-dimethyl-9,10-dihydrophenanthrene-5-carbaldehyde (**9**) (100MHz, $CDCl_3$) 25
Figure S37. ^{1}H-^{1}H COSY spectrum of 2-hydroxy-1,7-dimethyl-9,10-dihydrophenanthrene-5-carbaldehyde (**9**) (400MHz, $CDCl_3$) 26
Figure S38. ^{1}H-^{13}C HSQC spectrum of 2-hydroxy-1,7-dimethyl-9,10-dihydrophenanthrene-5-carbaldehyde (**9**) (400MHz, $CDCl_3$) 26
Figure S39. ^{1}H-^{13}C HMBC spectrum of 2-hydroxy-1,7-dimethyl-9,10-dihydrophenanthrene-5-carbaldehyde (**9**) (400MHz, $CDCl_3$) 27
Figure S40. HRESIMS of 2-hydroxy-1,7-dimethyl-9,10-dihydrophenanthrene-5-carbaldehyde (**9**) (negative ionisation mode) 27

Table 1. EDC data 19

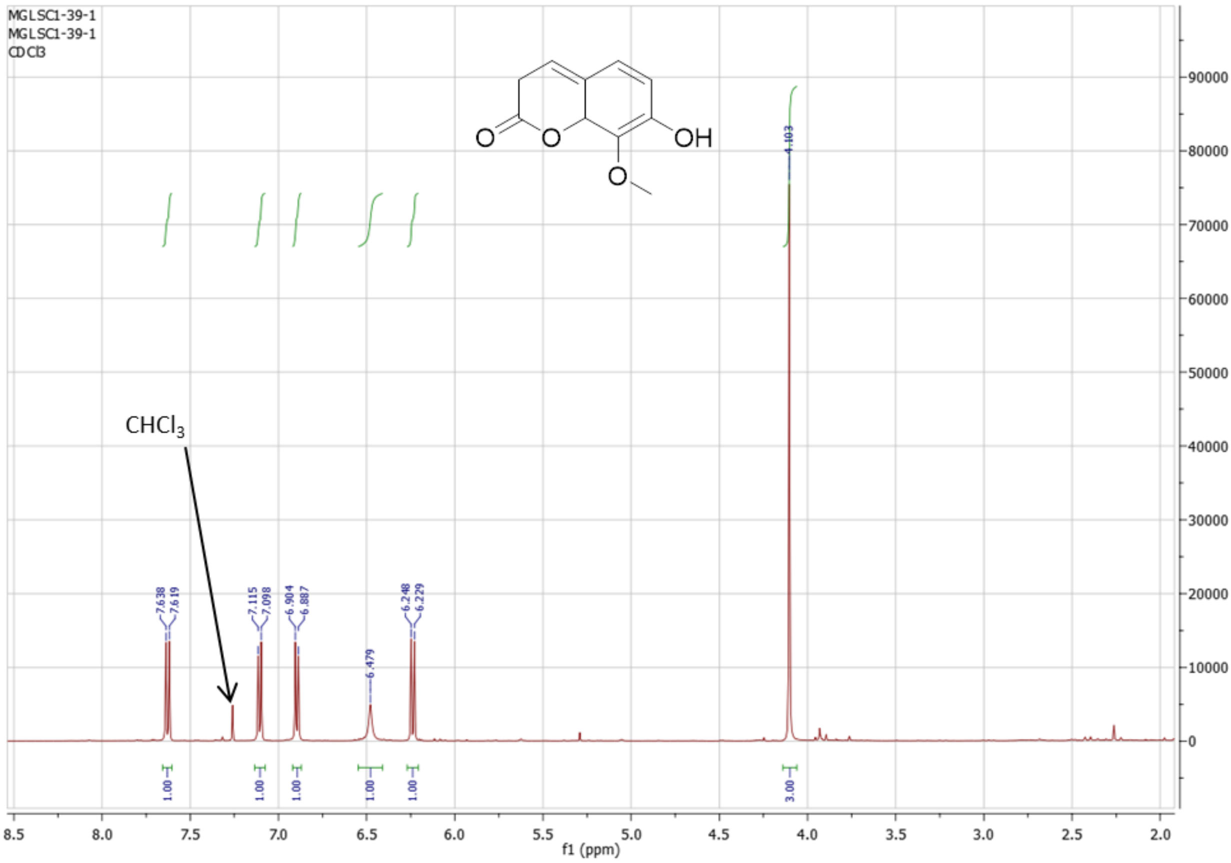

Figure S1. ^{1}H-NMR spectrum of hydrangetin (**1**) (500MHz, $CDCl_3$)

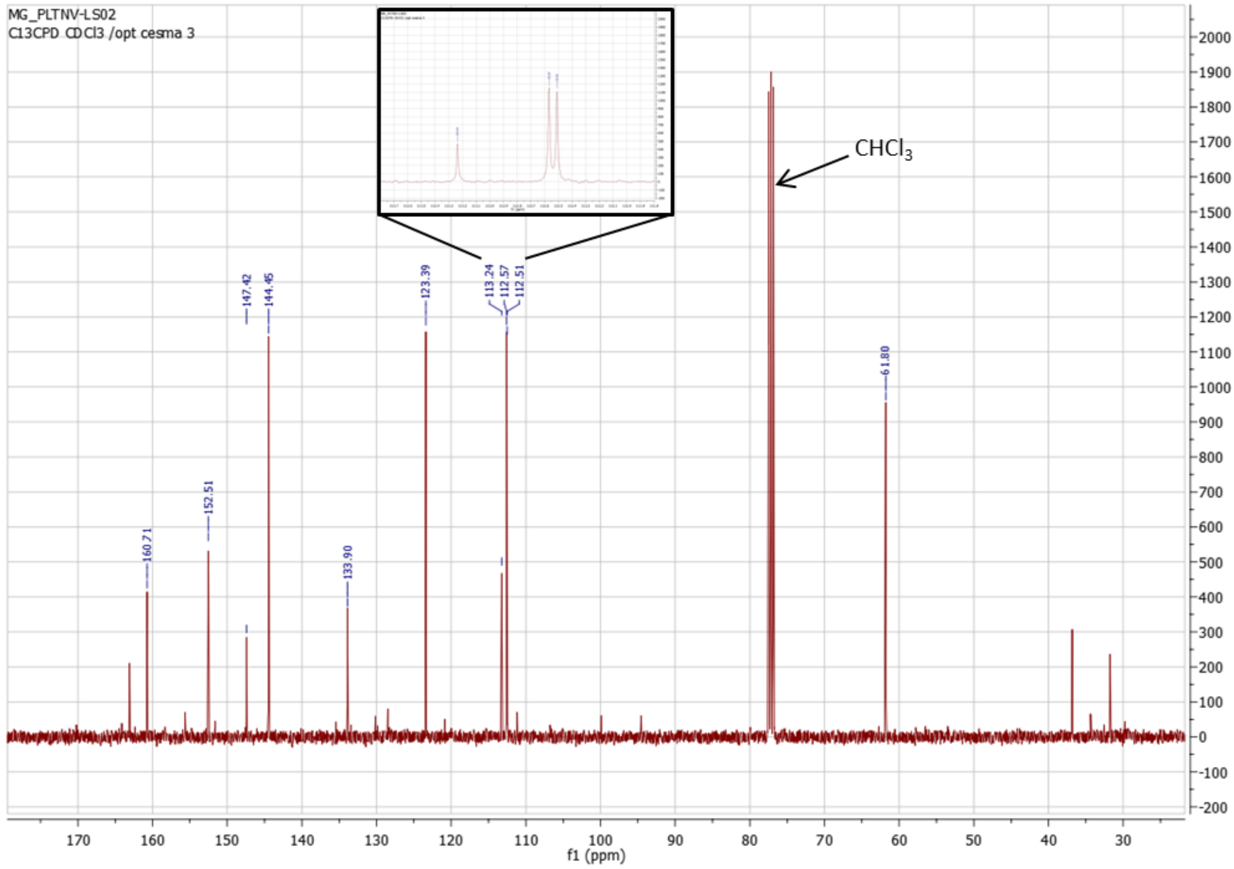

Figure S2. ^{13}C-NMR Spectrum of hydrangetin (**1**) (100MHz, $CDCl_3$)

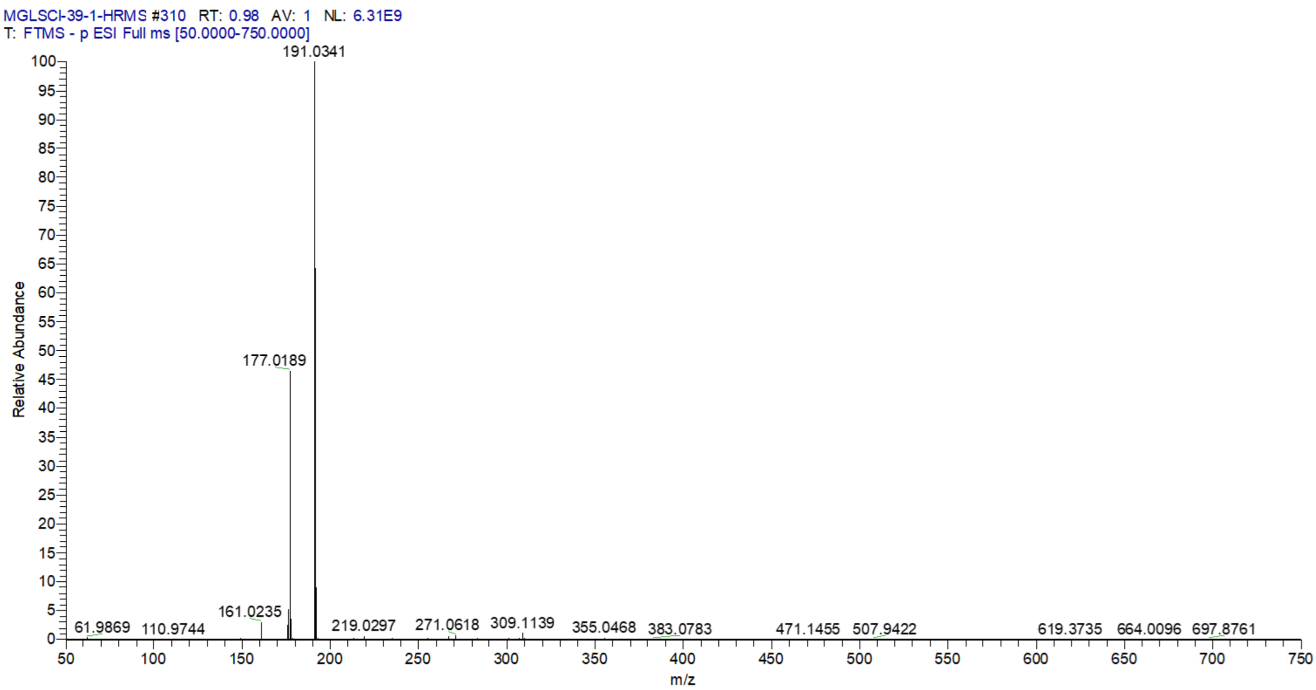

Figure S3. HRESIMS of hydrangetin (1) (negative ionisation mode)

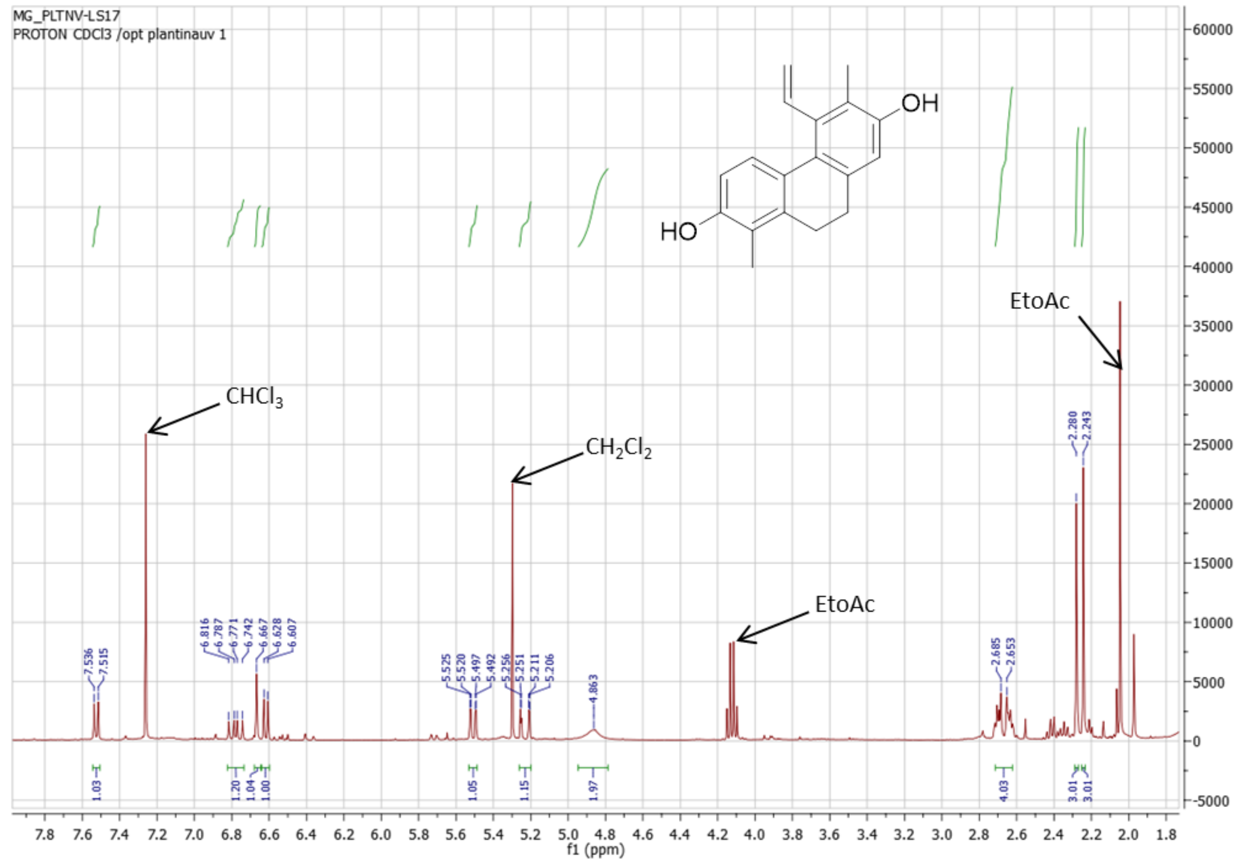

Figure S4. ^{1}H-NMR spectrum of juncusol (2) (400MHz, $CDCl_3$)

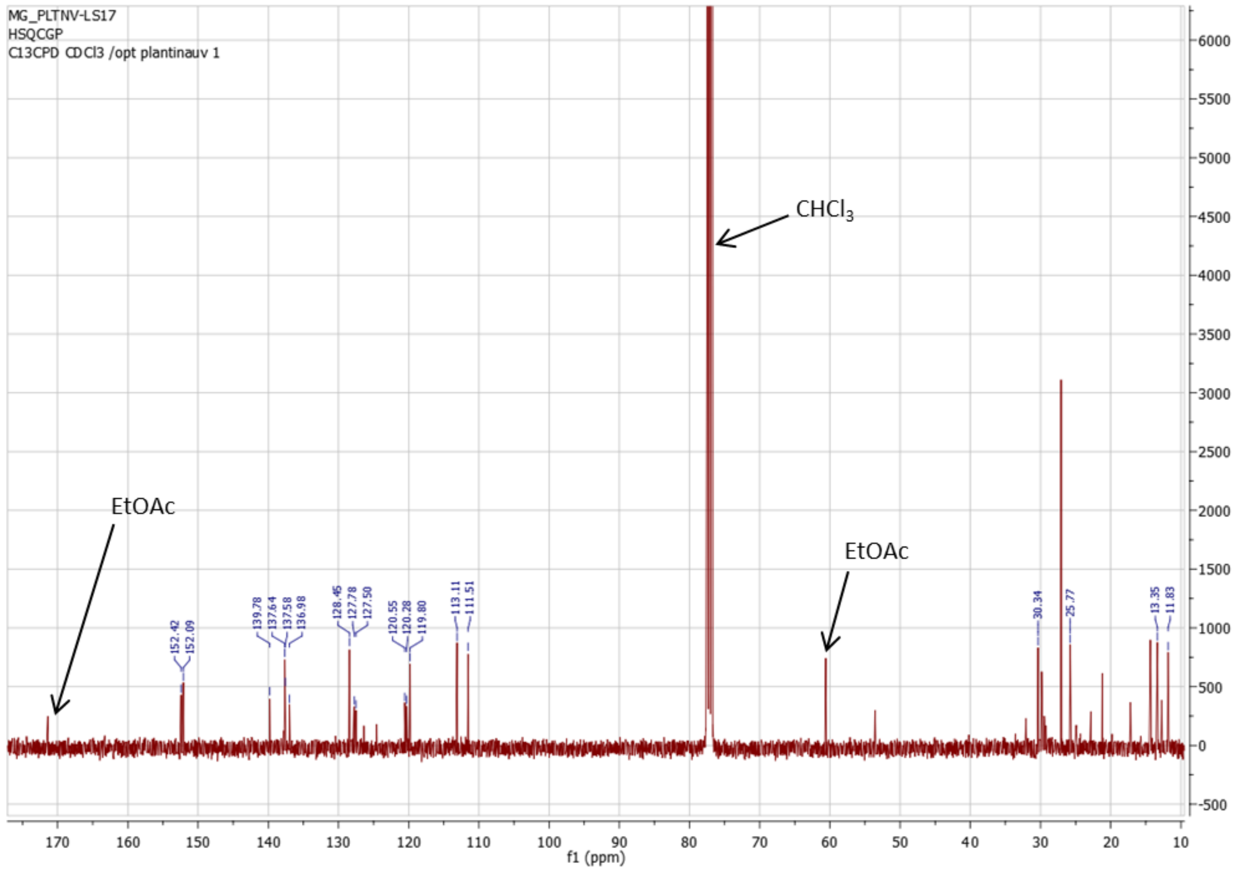

Figure S5. ^{13}C-NMR spectrum of juncusol (**2**) (100MHz, $CDCl_3$)

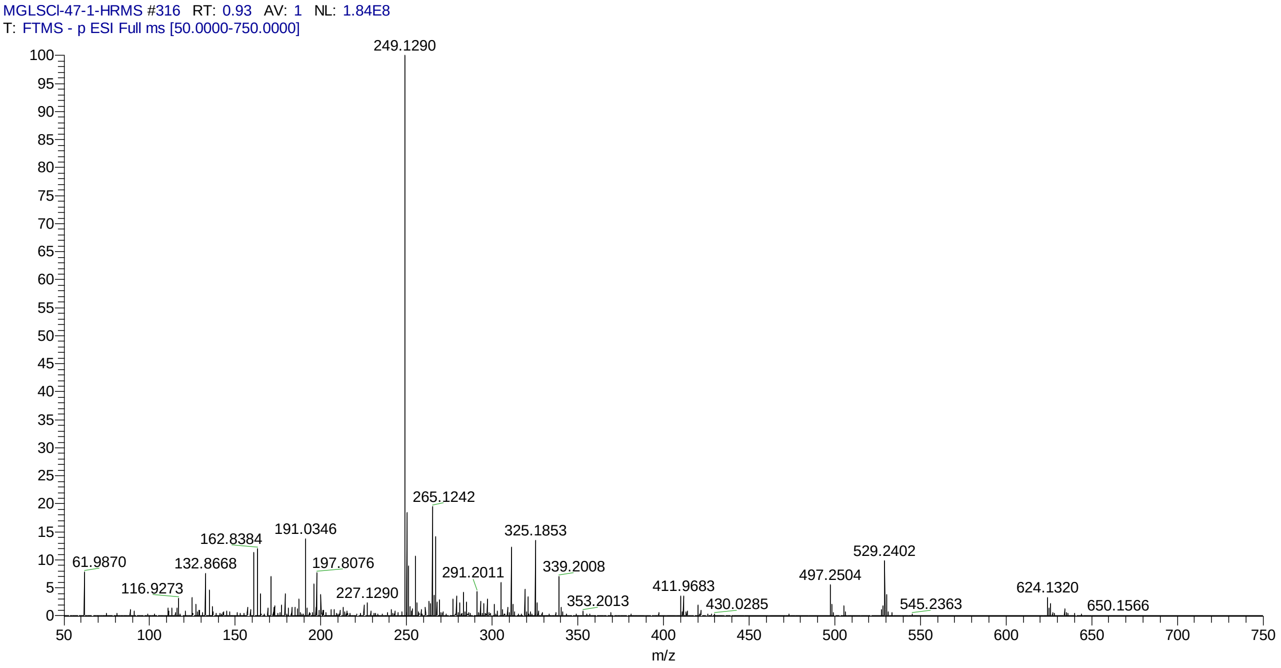

Figure S6. HRESIMS of juncusol (**2**) (negative ionisation mode)

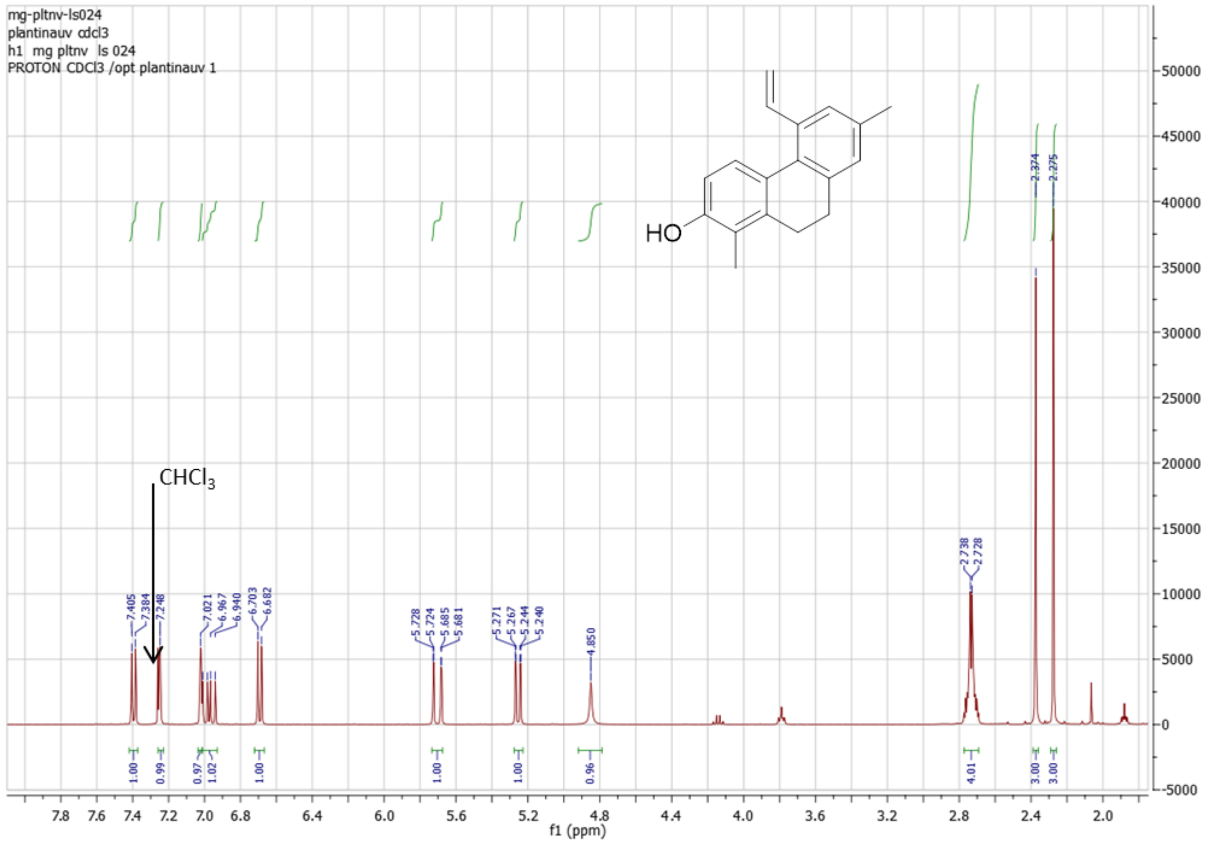

Figure S7. ^{1}H-NMR spectrum of juncunol (**3**) (400MHz, $CDCl_3$)

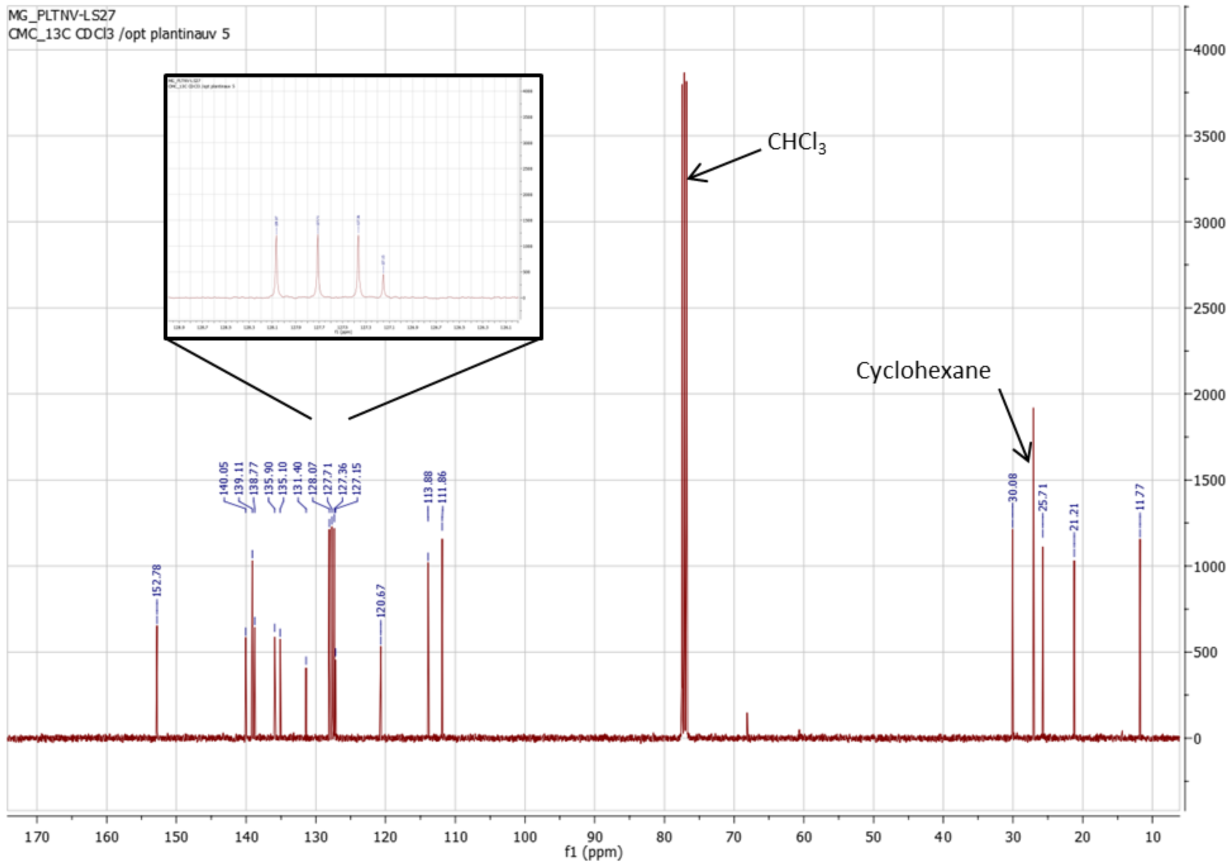

Figure S8. ^{13}C-NMR spectrum of juncunol (**3**) (100MHz, $CDCl_3$)

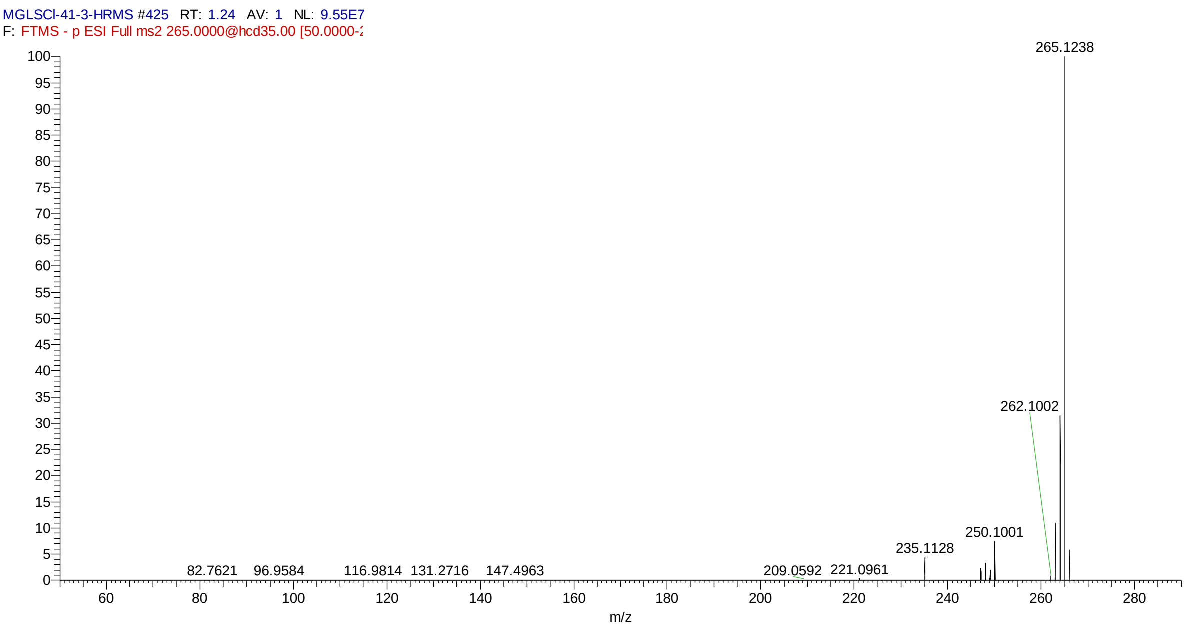

Figure S9. HRESIMS of juncunol (**3**) (negative ionisation mode)

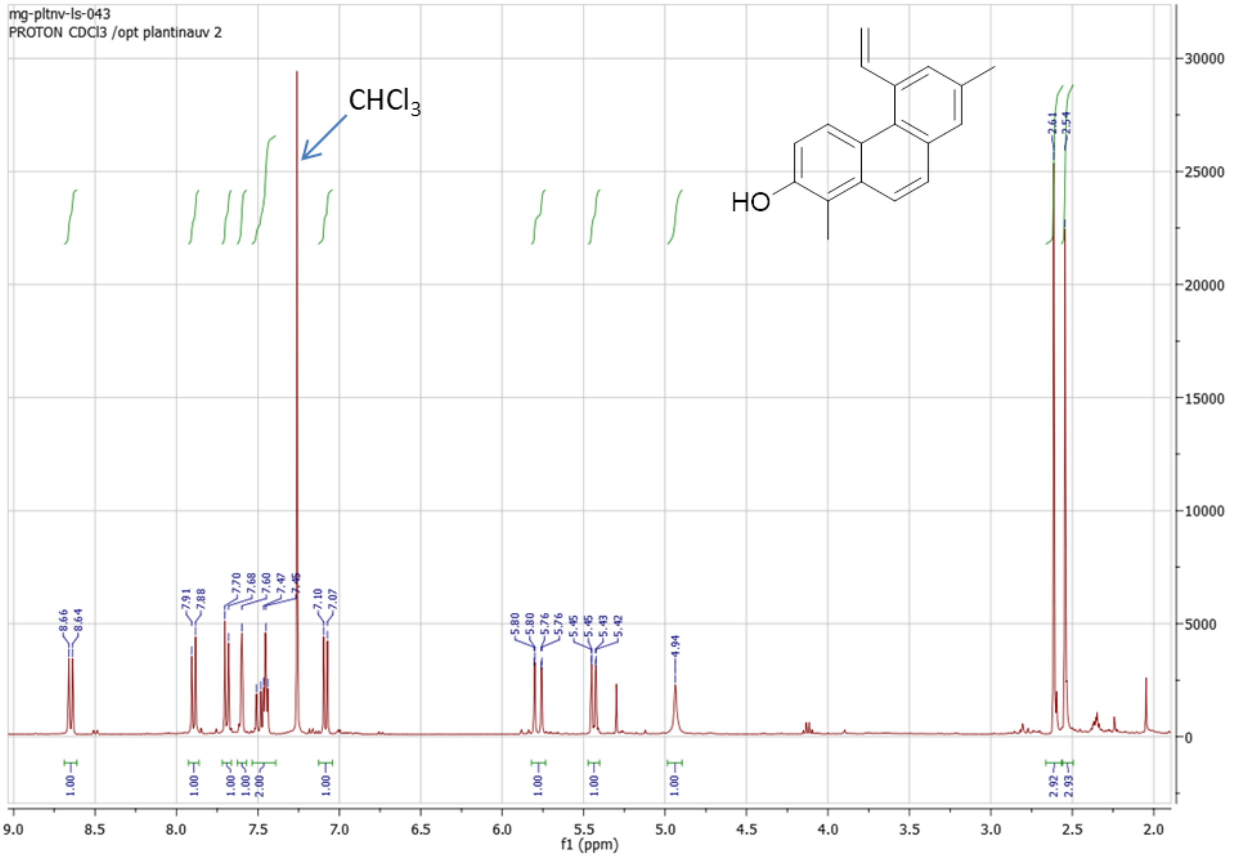

Figure S10. ^{1}H-NMR spectrum of 1,7-dimethyl-5-vinyl phenanthren-2-ol (**4**) (400MHz, $CDCl_3$)

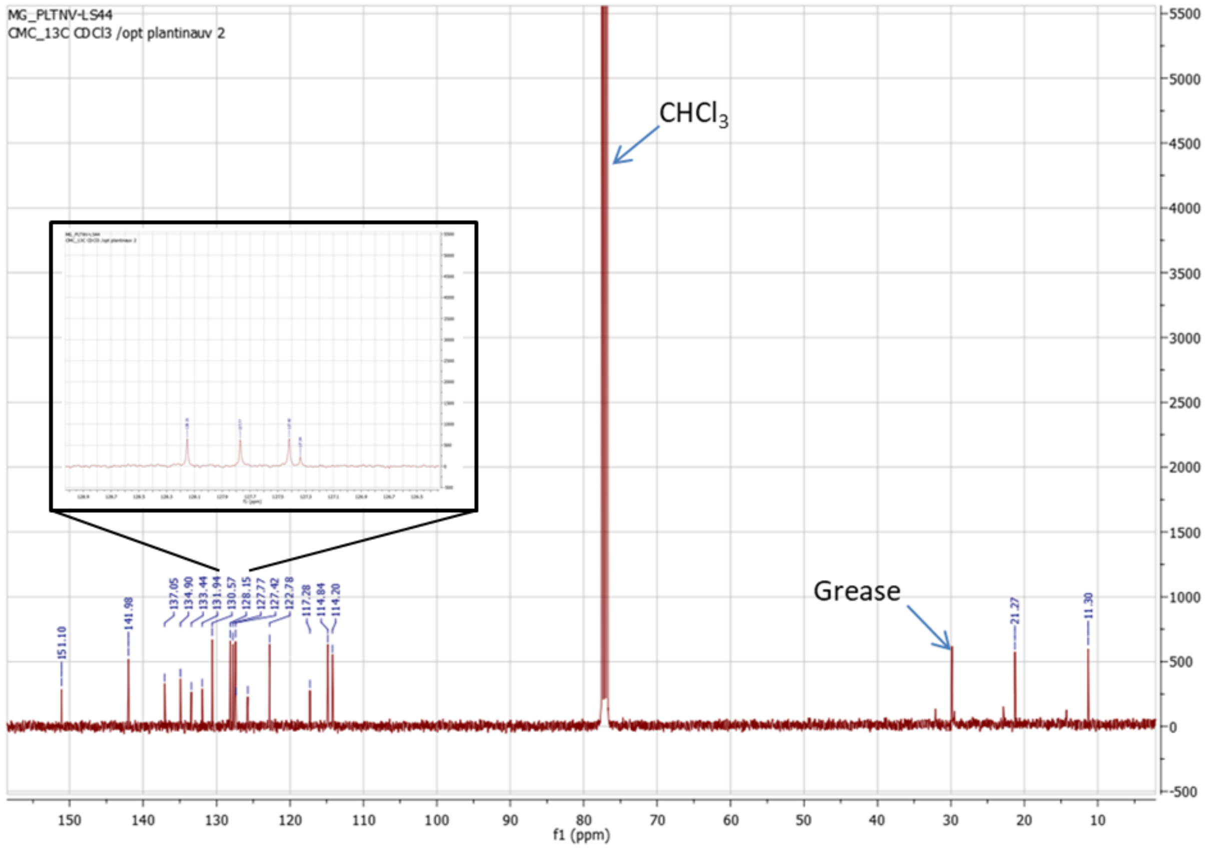

Figure S11. ^{13}C-NMR spectrum of 1,7-dimethyl-5-vinyl phenanthren-2-ol (**4**) (100MHz, CDCl$_3$)

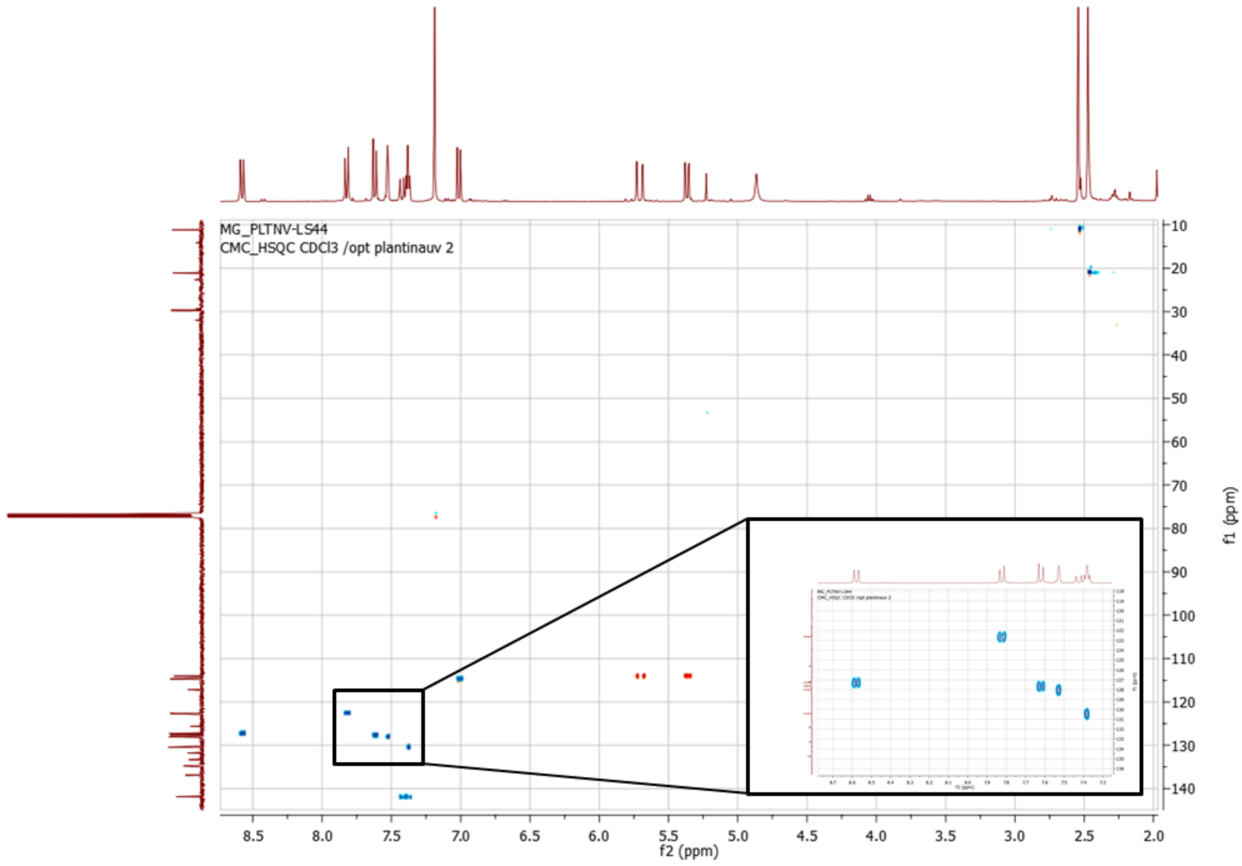

Figure S12. ^{1}H-^{13}C HSQC spectrum of 1,7-dimethyl-5-vinyl phenanthren-2-ol (**4**) (400MHz, CDCl$_3$)

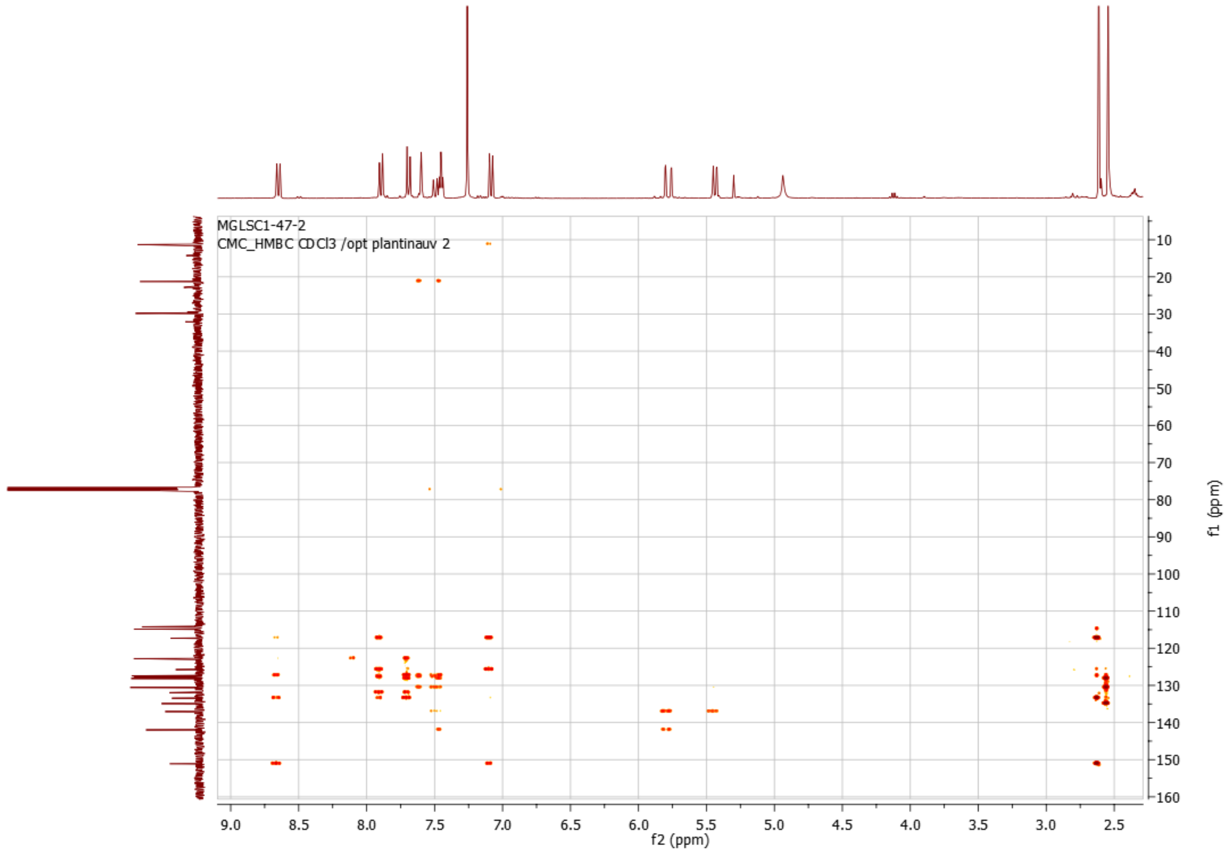

Figure S13. ^{1}H-^{13}C HMBC spectrum of 1,7-dimethyl-5-vinyl phenanthren-2-ol (**4**) (400MHz, $CDCl_3$)

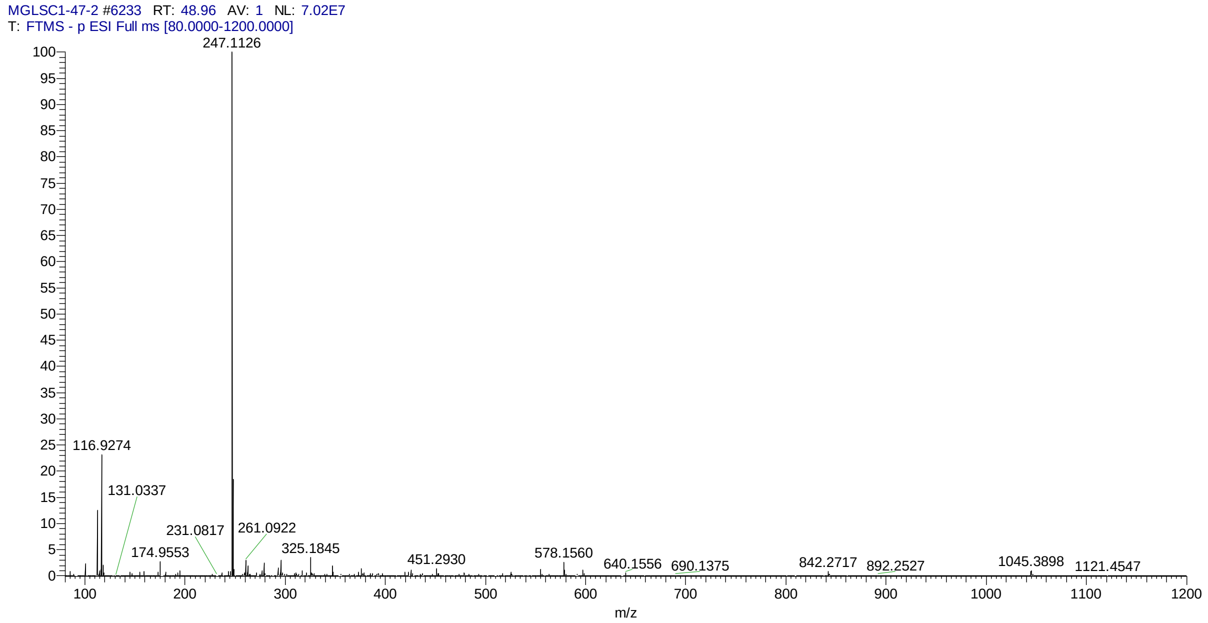

Figure S14. HRESIMS of 1,7-dimethyl-5-vinyl phenanthren-2-ol (**4**) (negative ionisation mode)

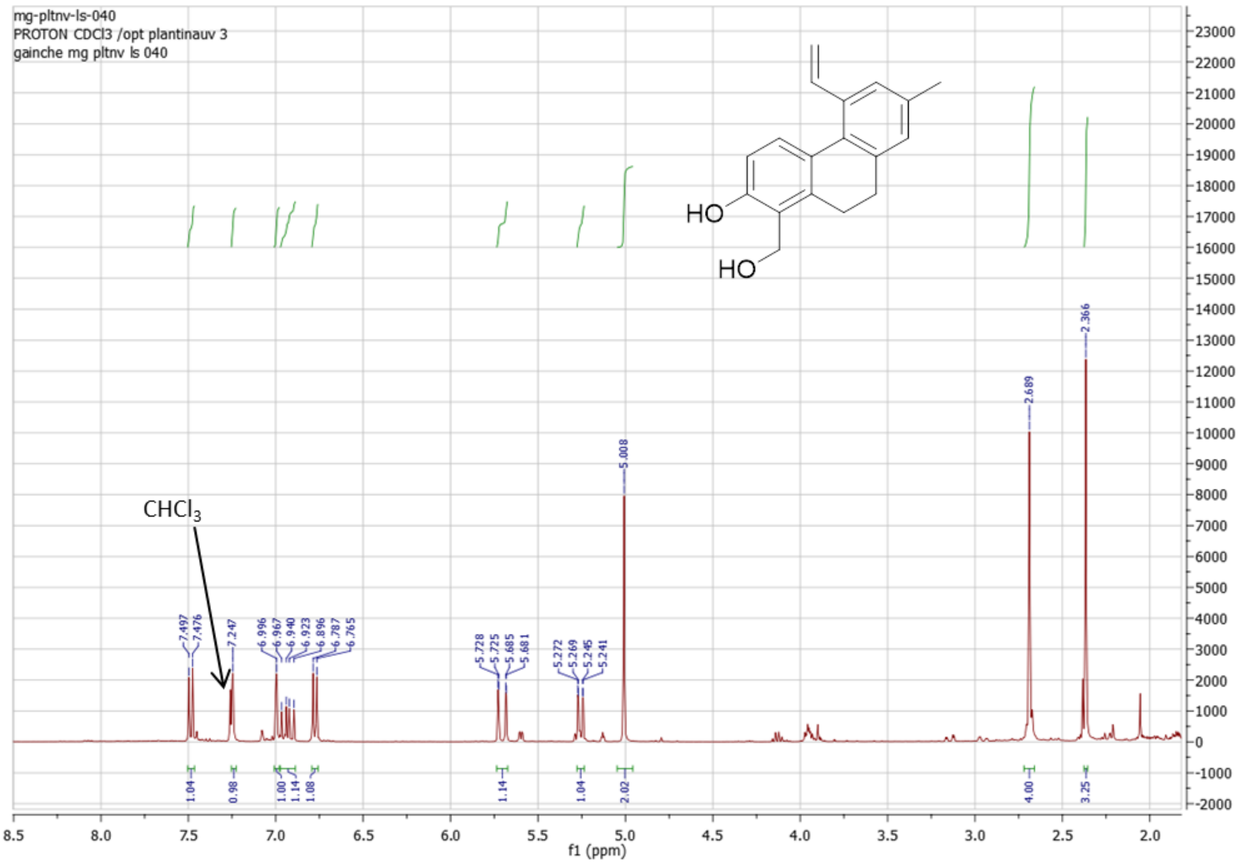

Figure S15. ^{1}H-NMR spectrum of 1-hydroxymethyl-7-methyl-5-vinyl-9,10-hydrophenanthren-2-ol (**5**) (400MHz, $CDCl_3$)

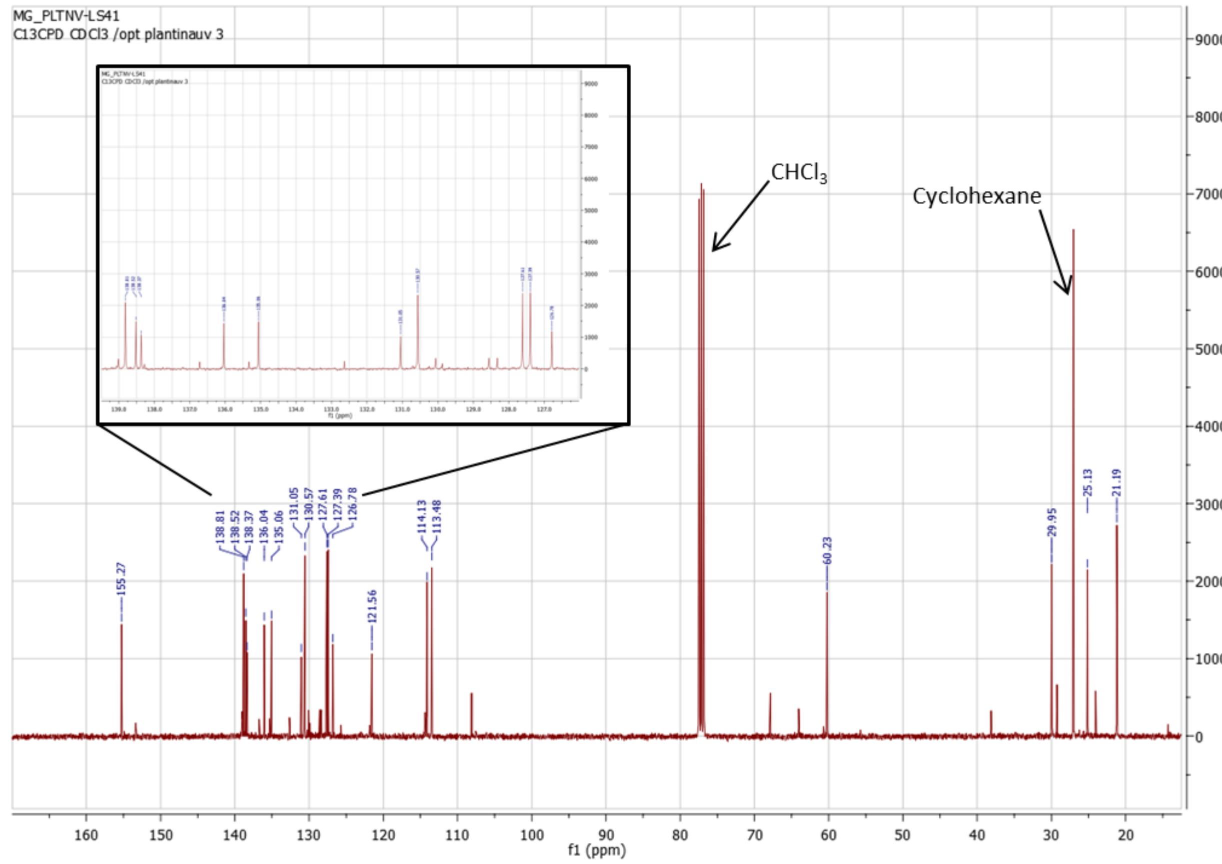

Figure S16. ^{13}C-NMR spectrum of 1-hydroxymethyl-7-methyl-5-vinyl-9,10-hydrophenanthren-2-ol (**5**) (100MHz, $CDCl_3$)

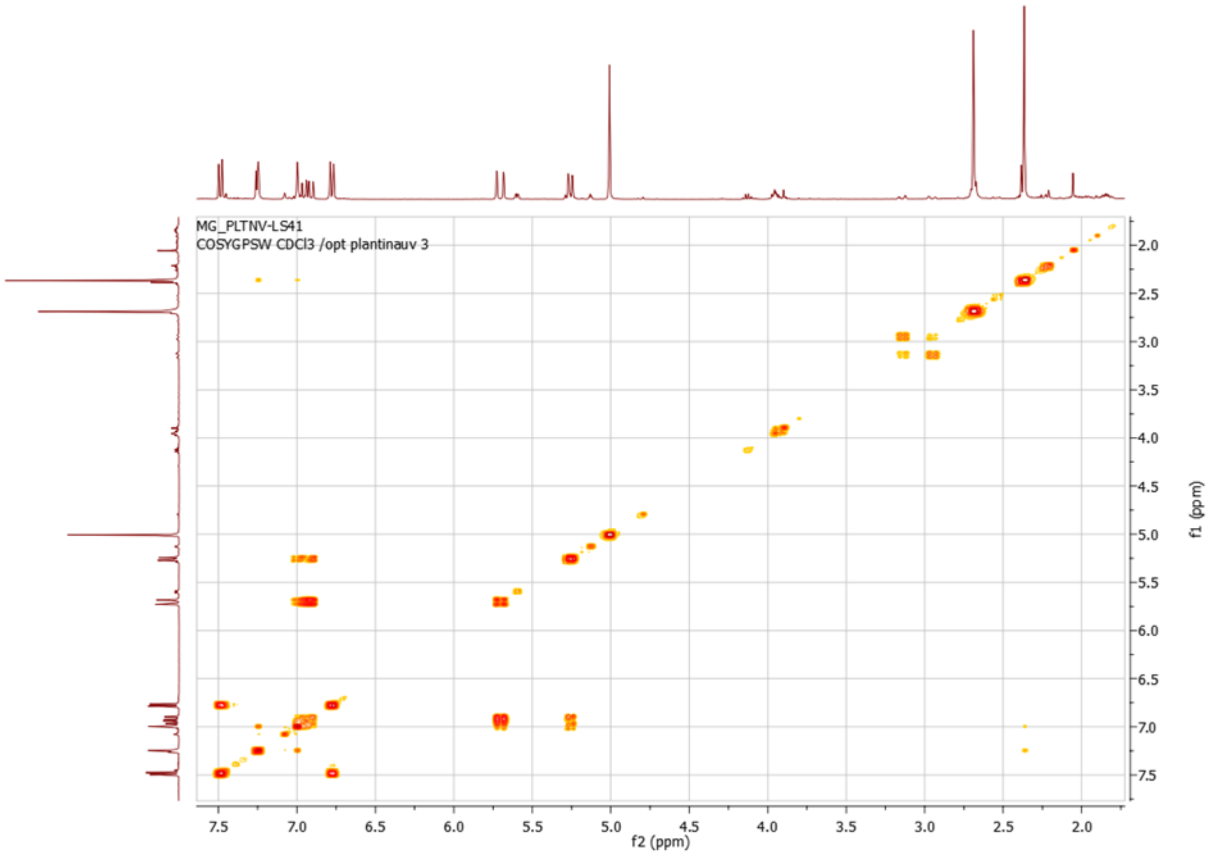

Figure S17. ^{1}H-^{1}H COSY spectrum of 1-hydroxymethyl-7-methyl-5-vinyl-9,10-hydrophenanthren-2-ol (**5**) (400MHz, $CDCl_3$)

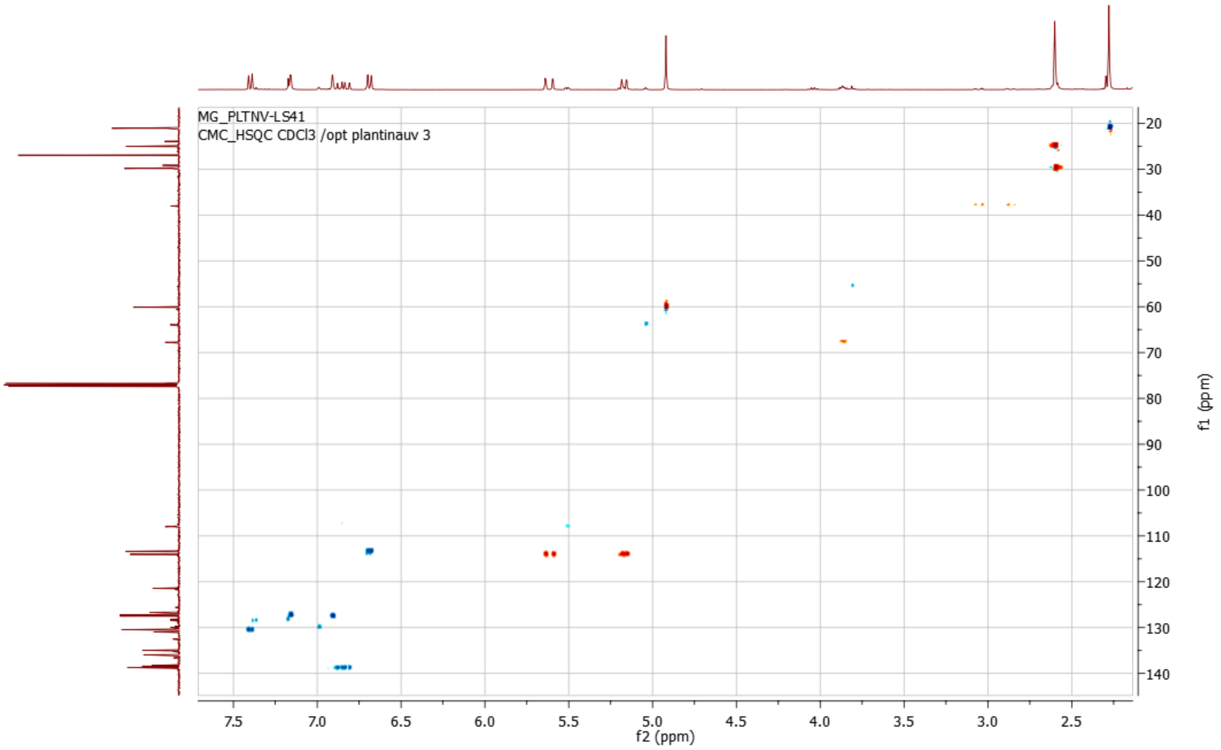

Figure S18. ^{1}H-^{13}C HSQC spectrum of 1-hydroxymethyl-7-methyl-5-vinyl-9,10-hydrophenanthren-2-ol (**5**) (400MHz, $CDCl_3$)

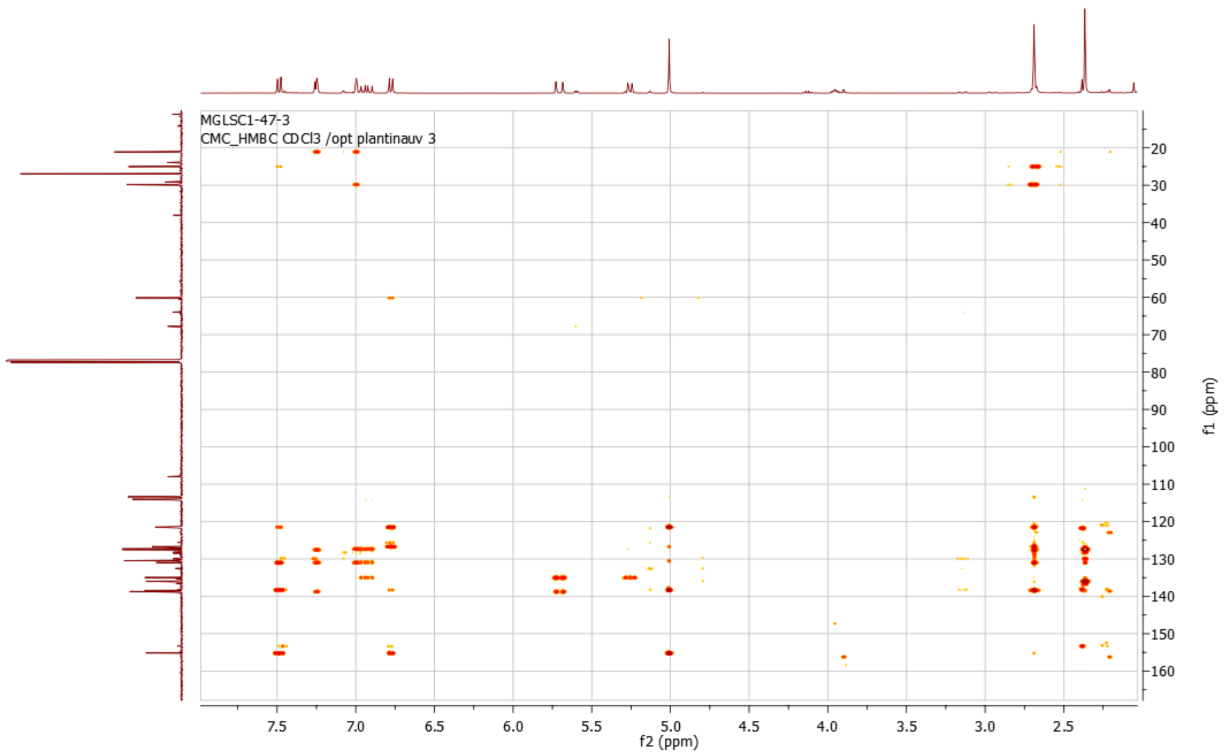

Figure S19. ^{1}H-^{13}C HMBC spectrum of 1-hydroxymethyl-7-methyl-5-vinyl-9,10-hydrophenanthren-2-ol (**5**) (400MHz, $CDCl_3$)

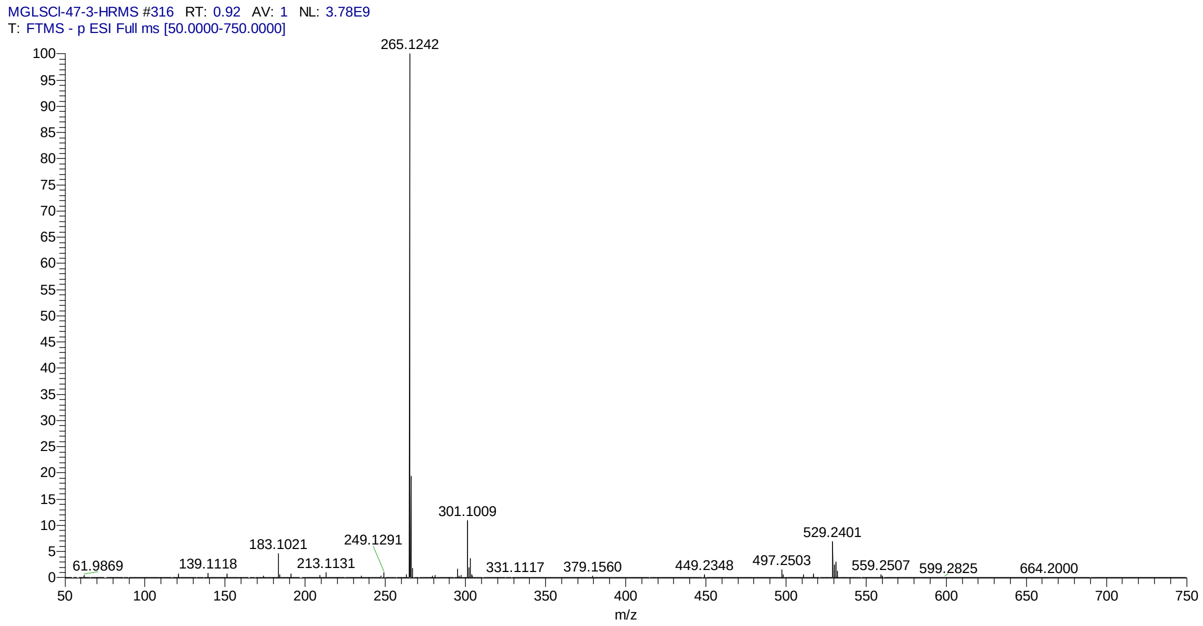

Figure S20. HRESIMS of 1-hydroxymethyl-7-methyl-5-vinyl-9,10-hydrophenanthren-2-ol (**5**) (negative ionisation mode)

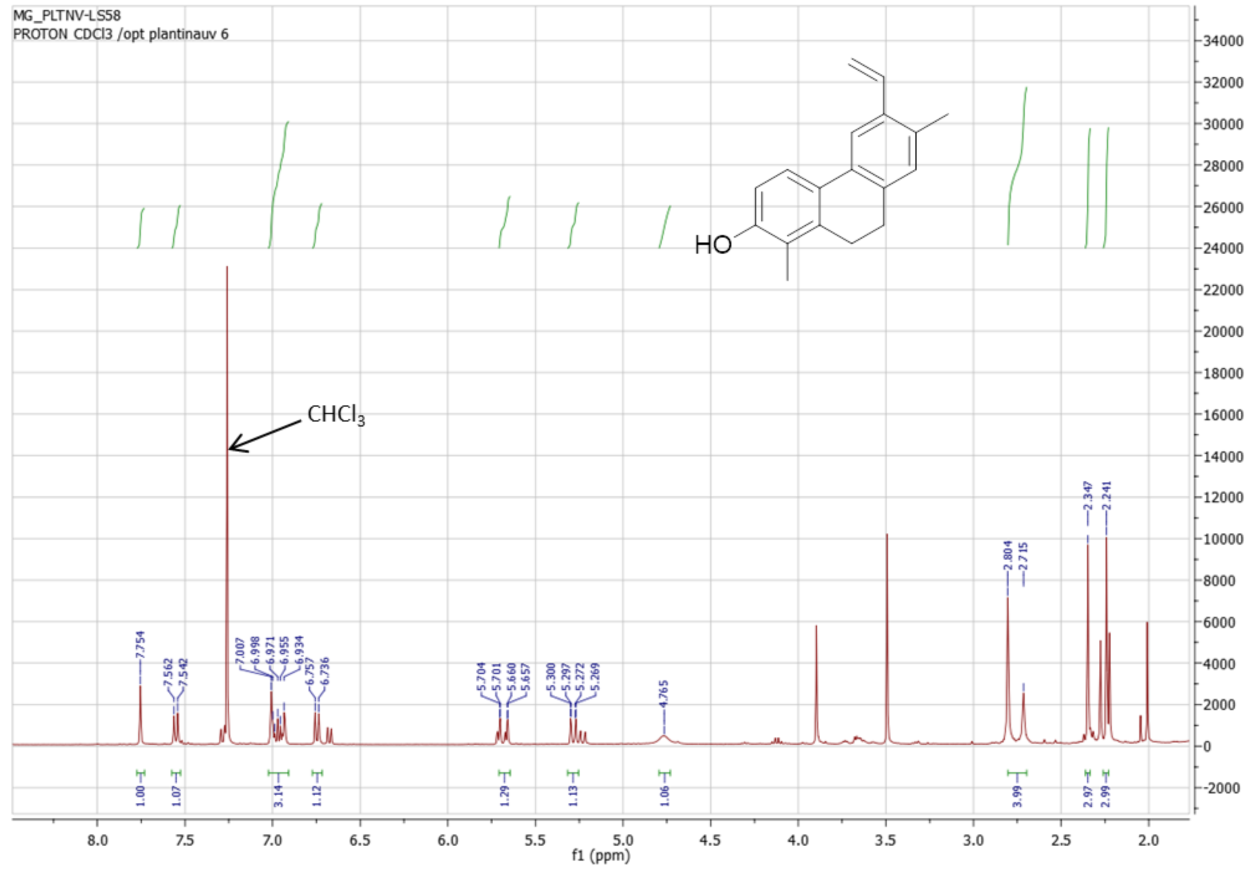

Figure S21. ^{1}H-NMR spectrum of juncuenin A (**6**) (400MHz, $CDCl_3$)

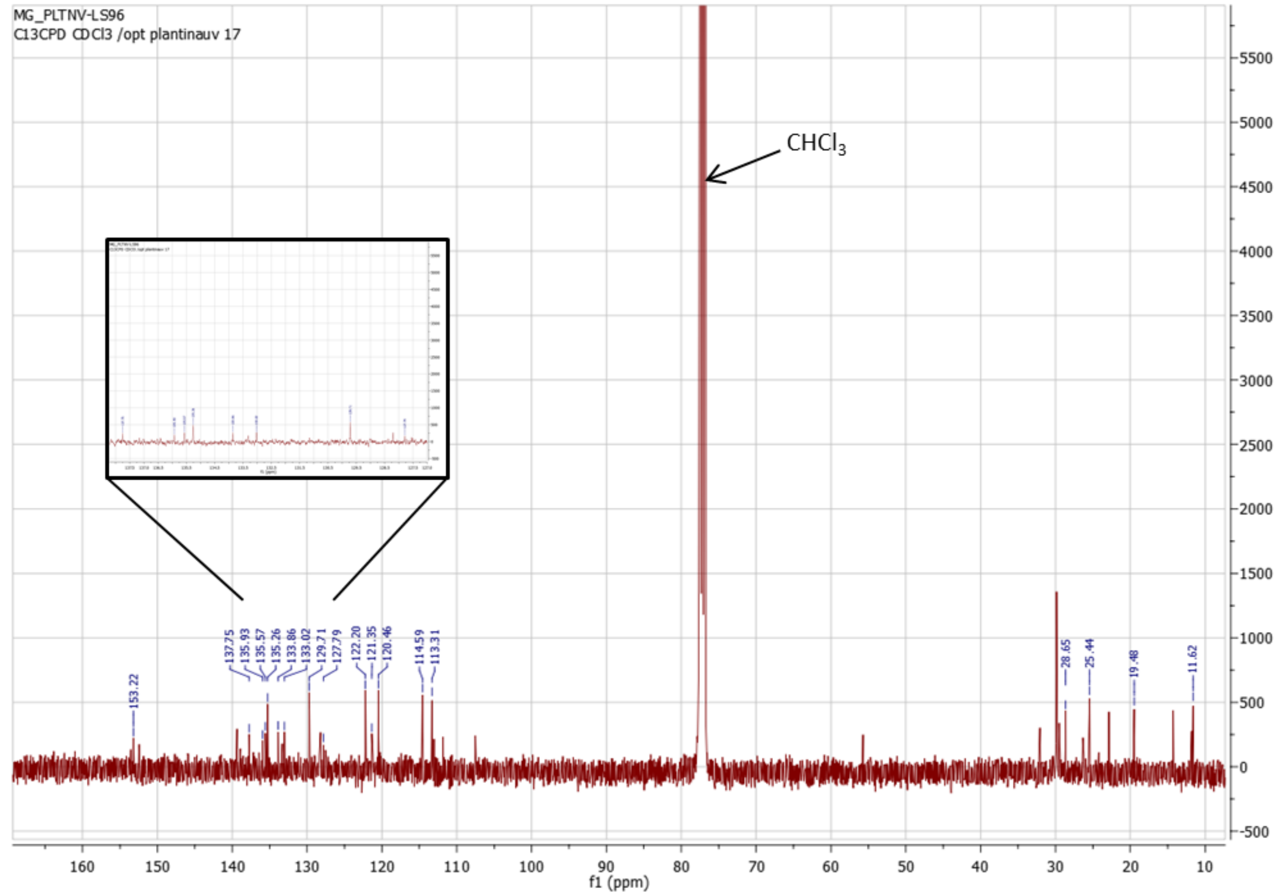

Figure S22. ^{13}C-NMR spectrum of juncuenin A (**6**) (100MHz, $CDCl_3$)

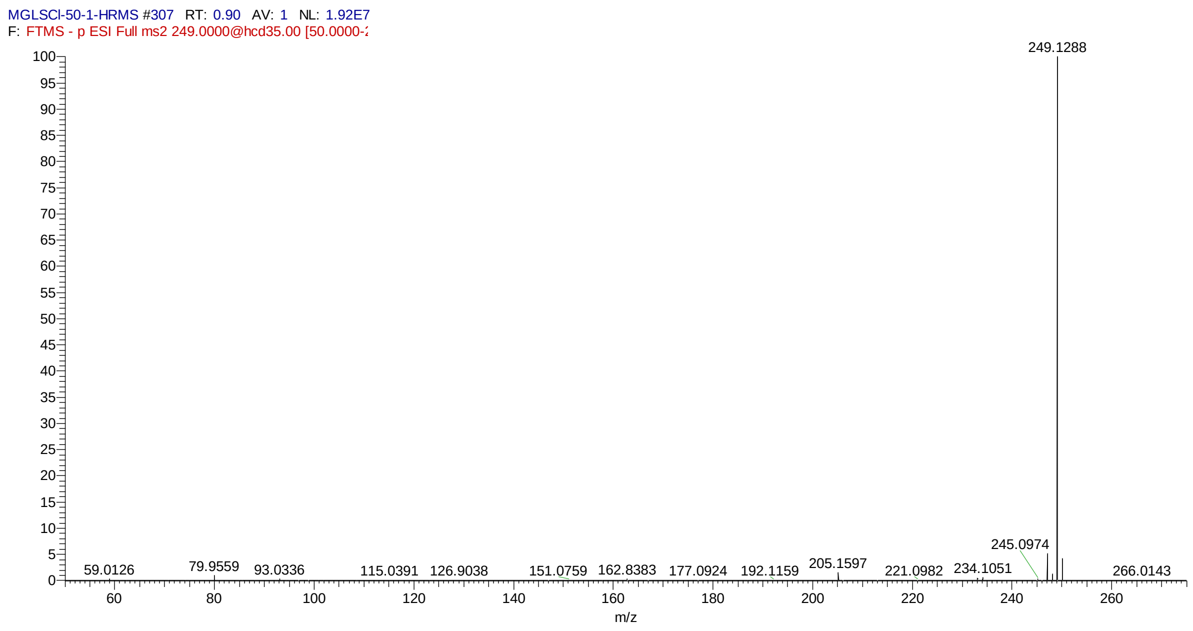

Figure S23. HRESIMS of juncuenin A (**6**) (negative ionisation mode)

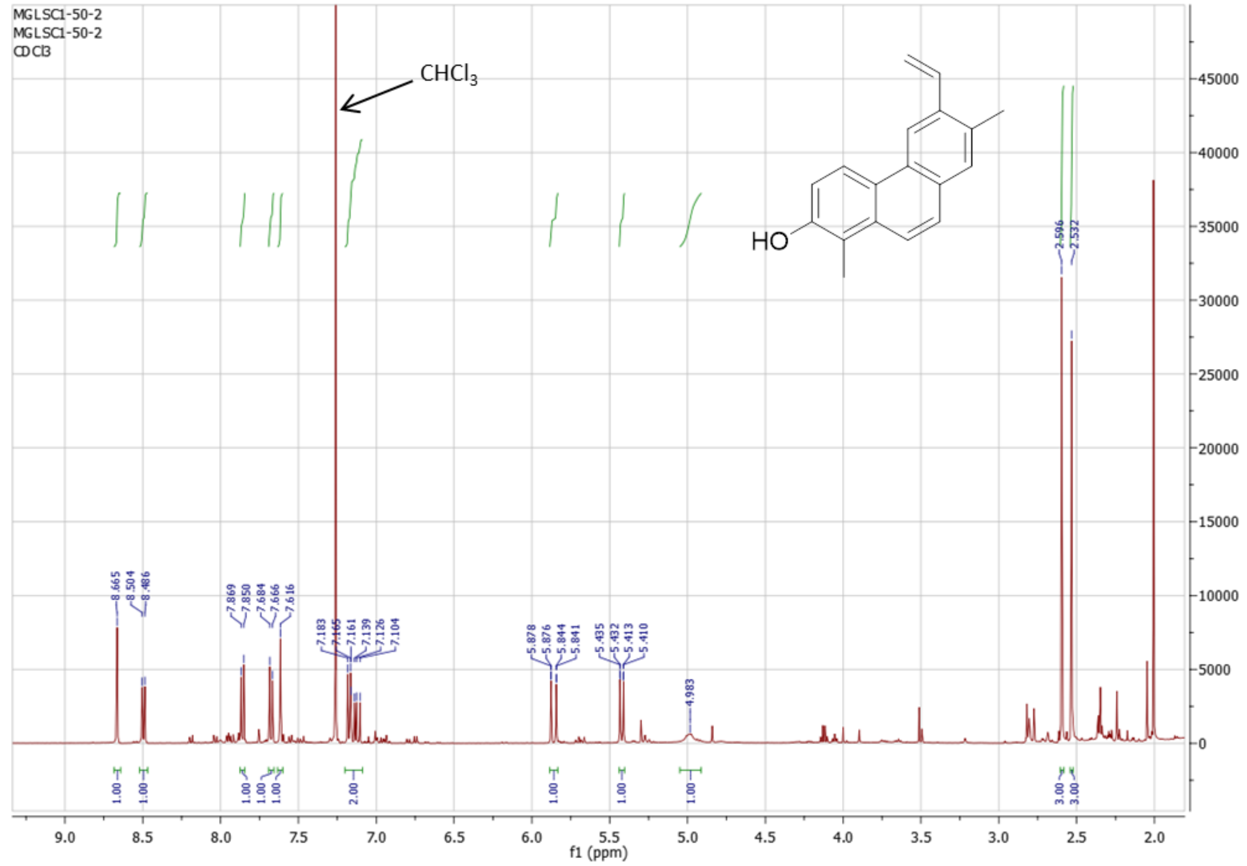

Figure S24. ^{1}H-NMR spectrum of dehydrojucuenin A (**7**) (400MHz, $CDCl_3$)

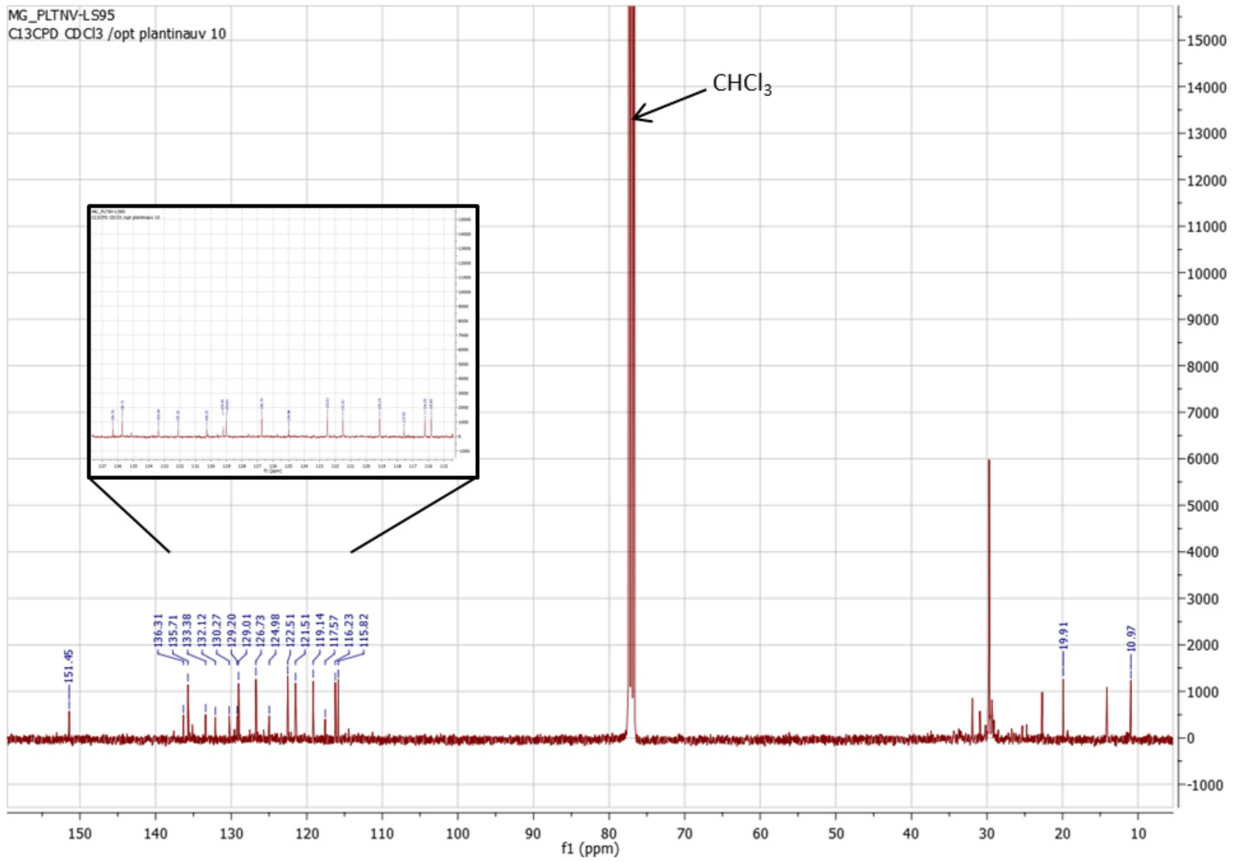

Figure S25. ^{13}C-NMR spectrum of dehydojuncuenin A (**7**) (100MHz, $CDCl_3$)

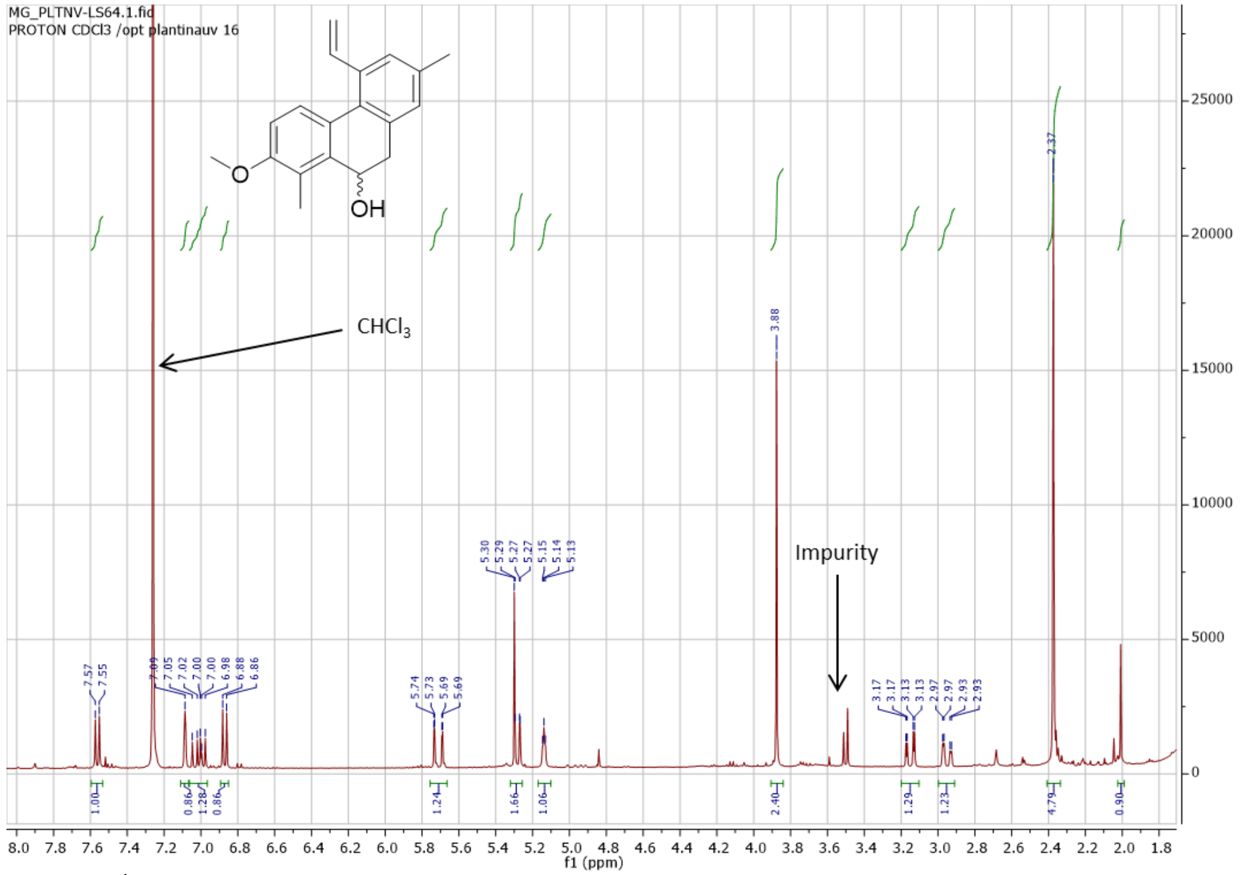

Figure S26. ^{1}H-NMR spectrum of 2-methoxy-1,7-dimethyl-5-vinyl-9,10-dihydrophenanthren-10-ol (**8**) (400MHz, $CDCl_3$)

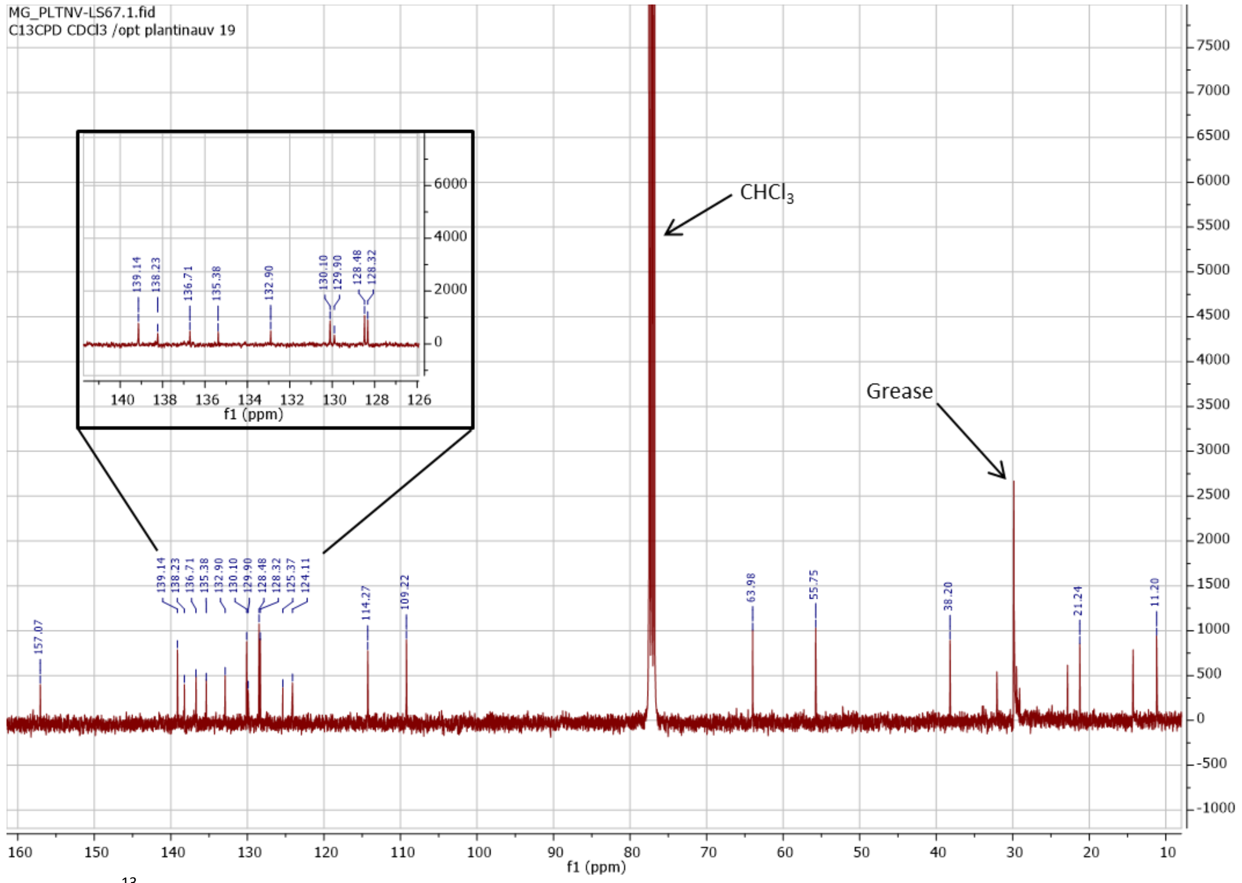

Figure S27. ^{13}C-NMR JMOD spectrum of 2-methoxy-1,7-dimethyl-5-vinyl-9,10-dihydrophenanthren-10-ol (**8**) (100MHz, $CDCl_3$, CH and CH_3 down, C and CH_2 up)

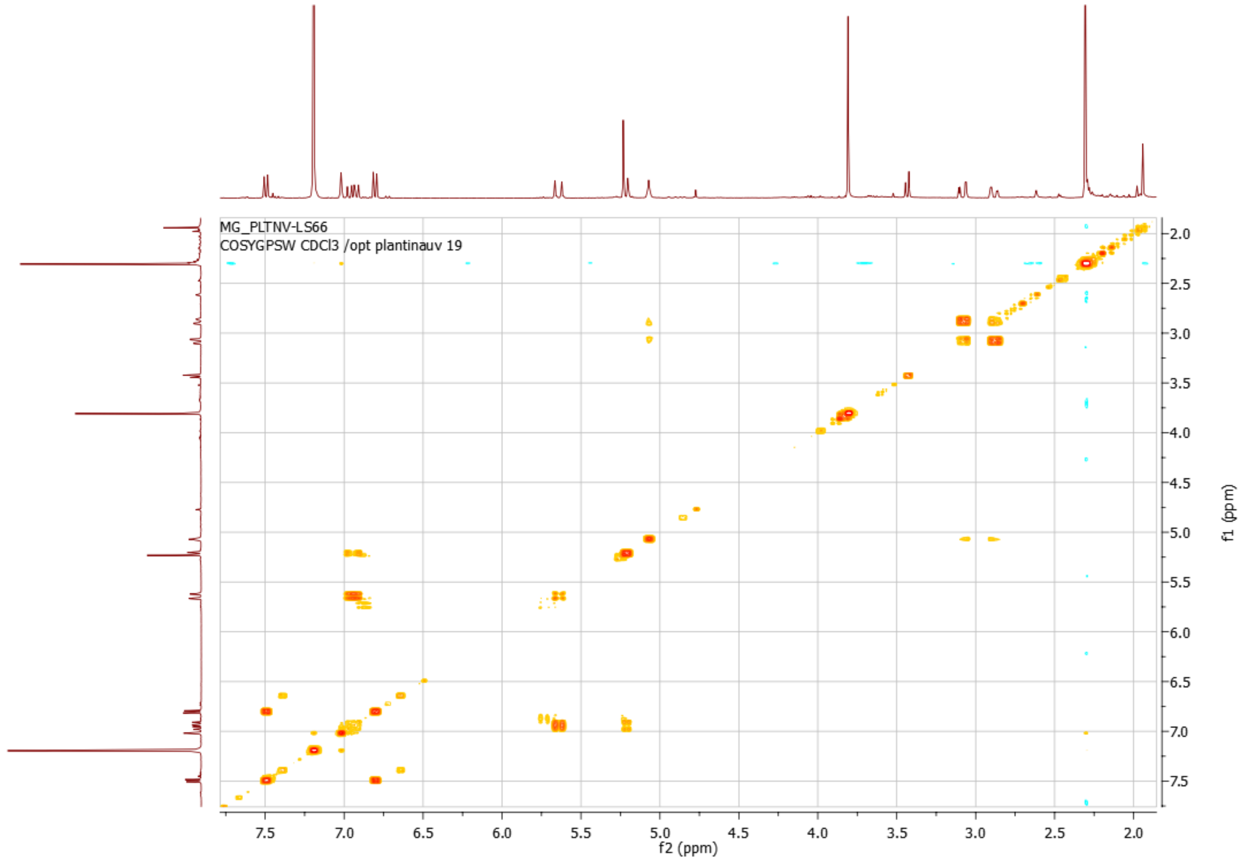

Figure S28. ^{1}H-^{1}H COSY spectrum of 2-methoxy-1,7-dimethyl-5-vinyl-9,10-dihydrophenanthren-10-ol (**8**) (400MHz, $CDCl_3$)

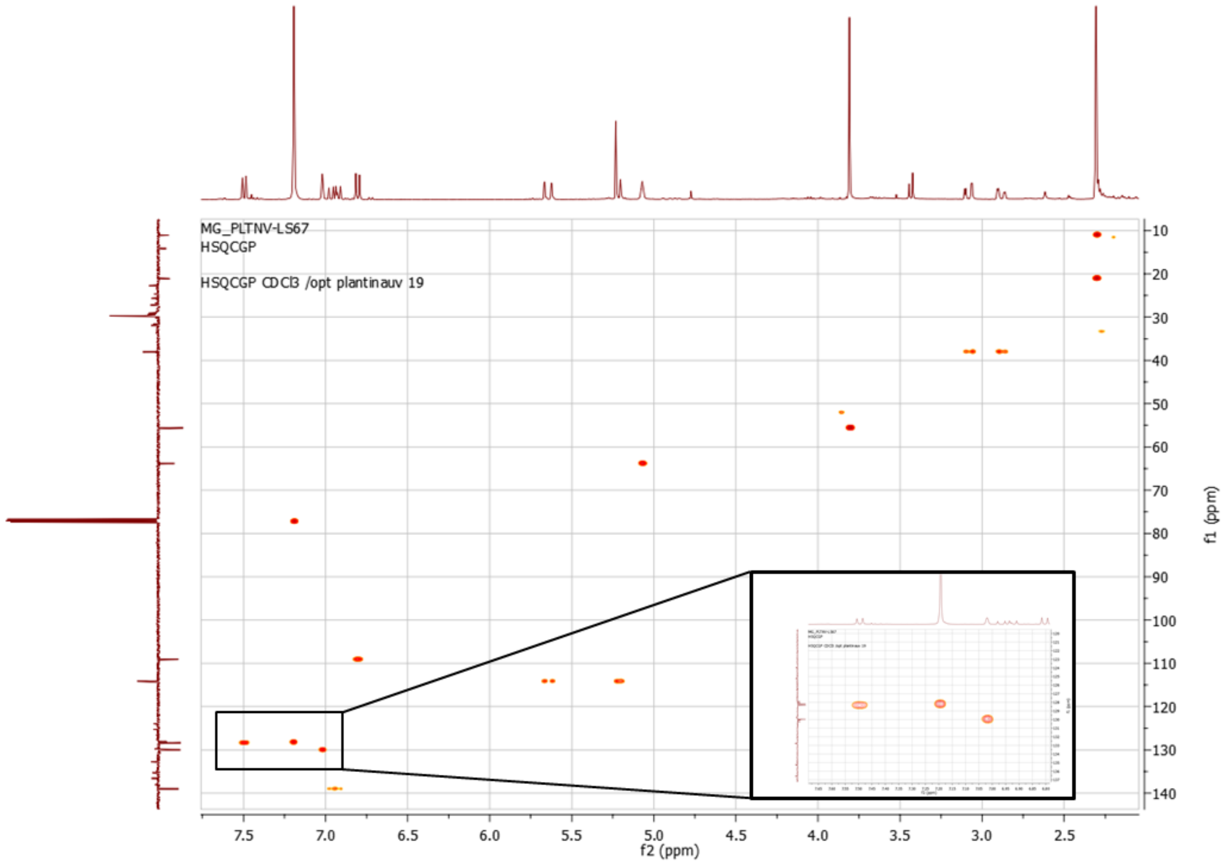

Figure S29. ^{1}H-^{13}C HSQC spectrum of 2-methoxy-1,7-dimethyl-5-vinyl-9,10-dihydrophenanthren-10-ol (**8**) (400MHz, $CDCl_3$)

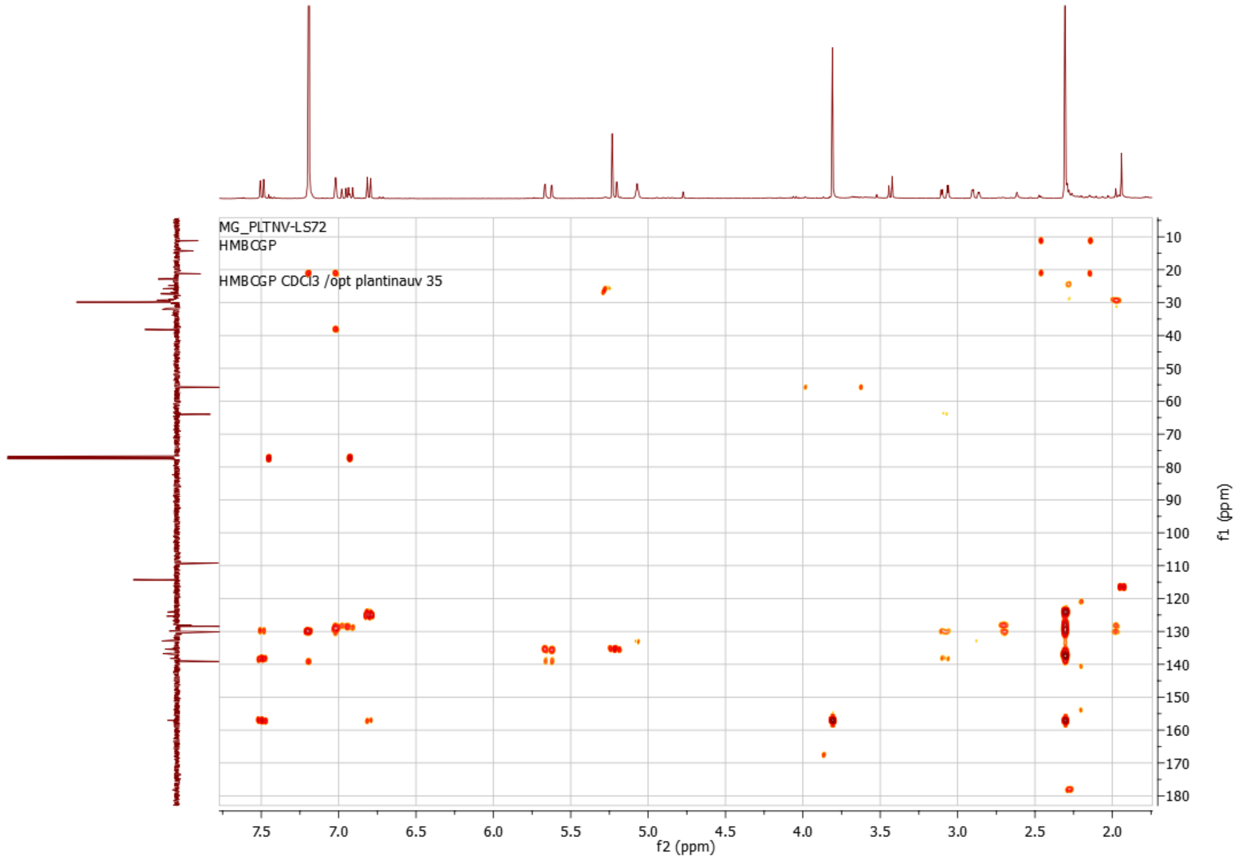

Figure S30. ^{1}H-^{13}C HMBC spectrum of 2-methoxy-1,7-dimethyl-5-vinyl-9,10-dihydrophenanthren-10-ol (**8**) (400MHz, $CDCl_3$)

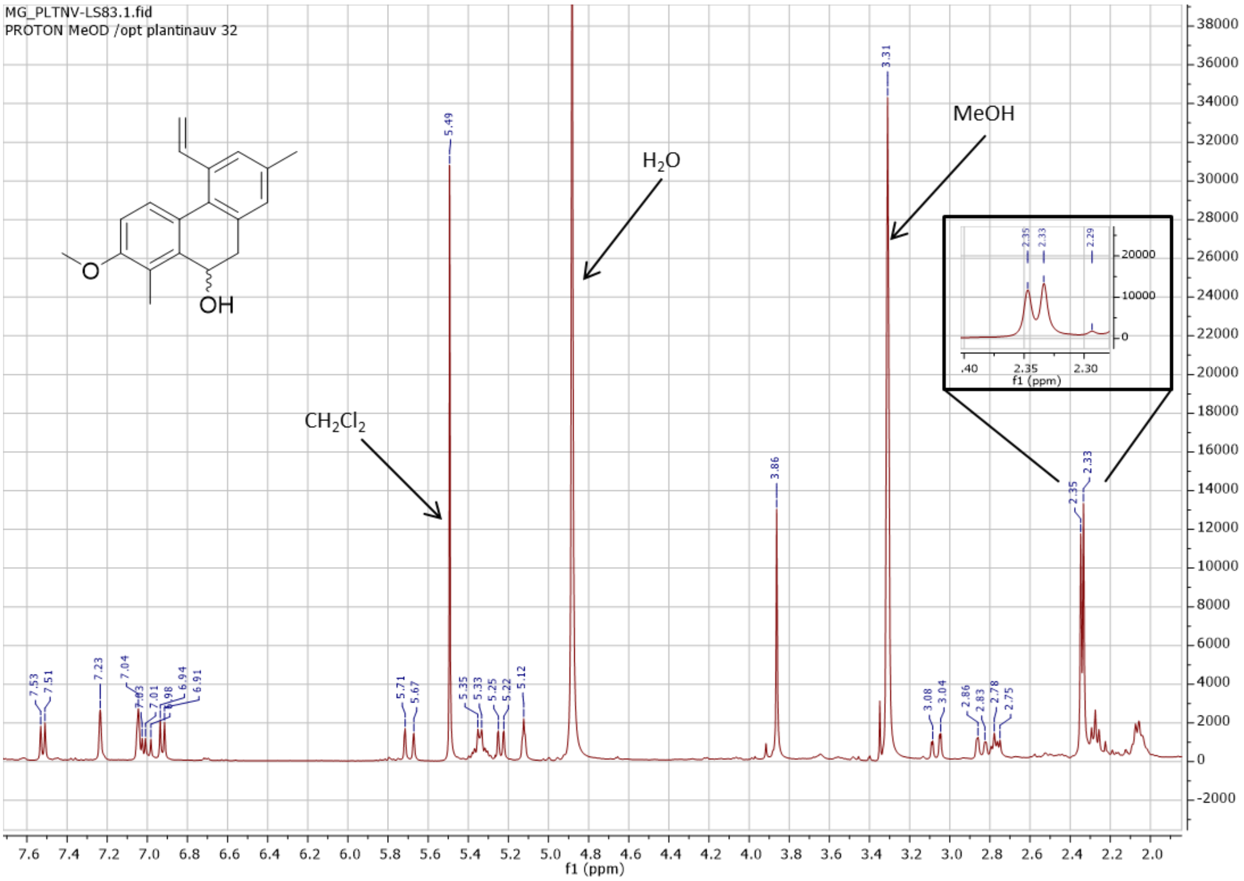

Figure S31. ^{1}H-NMR spectrum of 2-methoxy-1,7-dimethyl-5-vinyl-9,10-dihydrophenanthren-10-ol (8) (500MHz, CD_3OD)

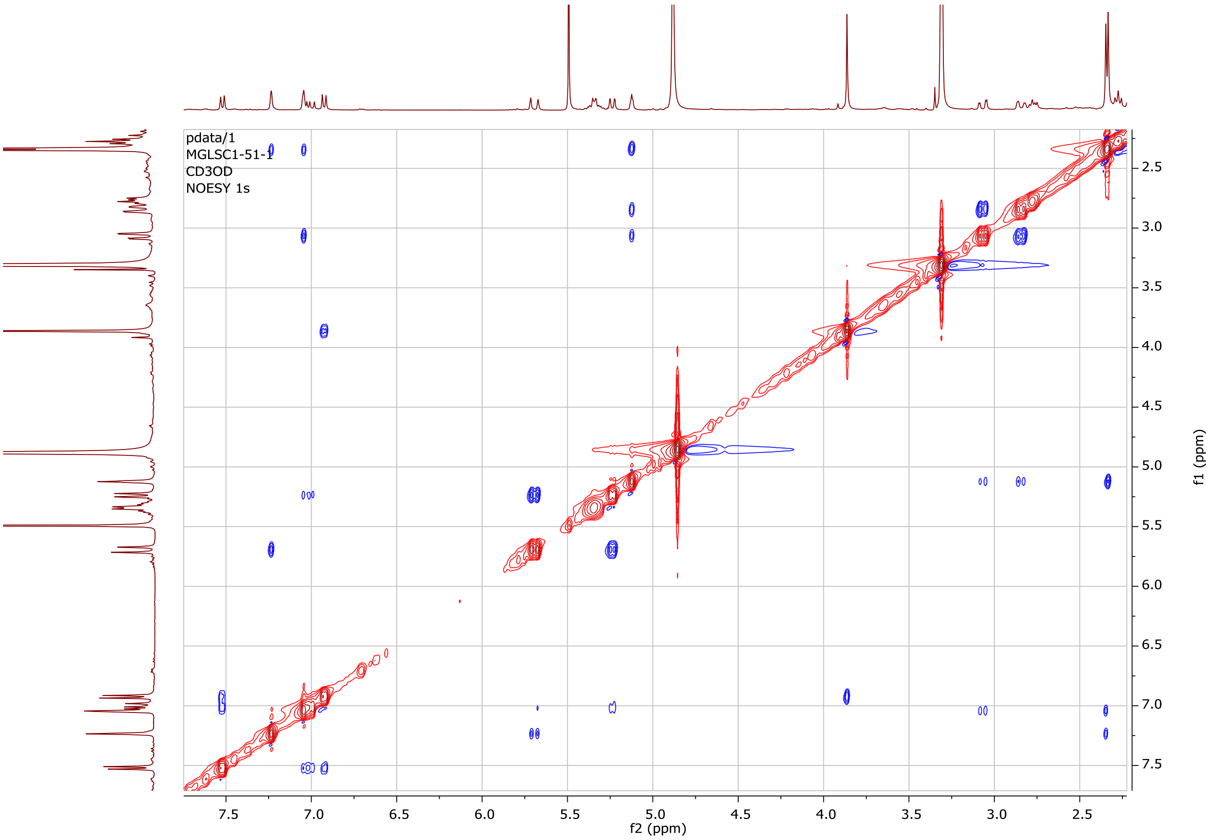

Figure S32. ^{1}H-^{1}H NOESY spectrum of 2-methoxy-1,7-dimethyl-5-vinyl-9,10-dihydrophenanthren-10-ol (8) (400MHz, CD_3OD)

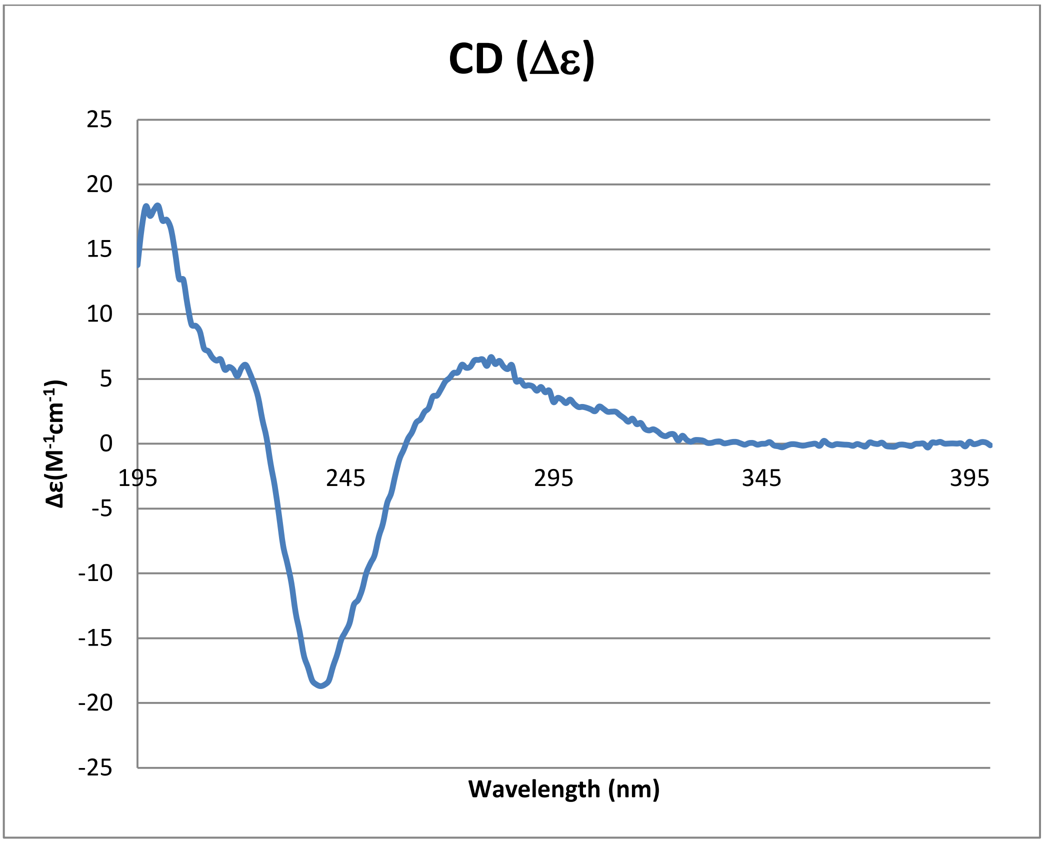

Figure S33. Experimental ECD spectra of compound 8

Circular Dichroism Data Analysis			
**Compound:**	*Compound 8*	Concentration [M]:	*0,0004*
**MW:**	500		
**Concentration in mg/mL:**	0,2	Formula:	[q] = (100 * q)/C*l
**Pathlength in cm:**	0,1		De = [q]/3298.2
**Wavelength (nm)**	**CD mdeg (!)**	**Molar ellipticity [θ] (deg cm^2 dmol^{-1})**	**CD (Δε) (mol^{-1} cm^{-1})**
**400**	-0,163119	-407,7975	-0,123642441
**399**	0,113959	284,8975	0,086379692
**398**	0,176983	442,4575	0,134151204
**397**	0,0305125	76,28125	0,023128146
**396**	-0,043186	-107,965	-0,032734522
**395**	0,201054	502,635	0,152396762
**394**	-0,272805	-682,0125	-0,206783245
**393**	0,0376598	94,1495	0,028545722
**392**	0,0113136	28,284	0,008575587

**391**	0,0335042	83,7605	0,025395822
**390**	0,0172675	43,16875	0,013088579
**389**	0,00959835	23,995875	0,007275446
**388**	0,190505	476,2625	0,144400734
**387**	0,0958515	239,62875	0,072654402
**386**	0,121044	302,61	0,091750045
**385**	-0,388797	-971,9925	-0,294703929
**384**	0,0141095	35,27375	0,010694849
**383**	0,00168215	4,205375	0,001275052
**382**	-0,025767	-64,4175	-0,019531108
**381**	-0,239674	-599,185	-0,181670305
**380**	-0,159702	-399,255	-0,121052392
**379**	-0,0845144	-211,286	-0,064061003
**378**	-0,114643	-286,6075	-0,086898157
**377**	-0,301934	-754,835	-0,228862713
**376**	-0,29269	-731,725	-0,221855861
**375**	-0,228754	-571,885	-0,173393063
**374**	0,108948	272,37	0,082581408
**373**	-0,0282365	-70,59125	-0,021402962
**372**	0,0268681	67,17025	0,02036573
**371**	0,134921	337,3025	0,102268662
**370**	-0,285939	-714,8475	-0,216738676
**369**	-0,154974	-387,435	-0,117468619
**368**	-0,0294795	-73,69875	-0,022345143
**367**	-0,210923	-527,3075	-0,159877357
**366**	-0,117492	-293,73	-0,089057668
**365**	-0,0952536	-238,134	-0,072201201
**364**	-0,061256	-153,14	-0,046431387
**363**	-0,0375537	-93,88425	-0,028465299
**362**	-0,167709	-419,2725	-0,127121612
**361**	-0,0115339	-28,83475	-0,008742572
**360**	0,295626	739,065	0,224081317
**359**	-0,218265	-545,6625	-0,165442514
**358**	0,00193203	4,830075	0,001464458
**357**	-0,0403899	-100,97475	-0,030615108
**356**	-0,119997	-299,9925	-0,090956431
**355**	-0,197973	-494,9325	-0,150061397
**354**	-0,132357	-330,8925	-0,100325177
**353**	-0,0485545	-121,38625	-0,036803787
**352**	-0,0580711	-145,17775	-0,044017267
**351**	-0,196139	-490,3475	-0,148671245
**350**	-0,360191	-900,4775	-0,27302089
**349**	-0,261429	-653,5725	-0,19816036
**348**	-0,170663	-426,6575	-0,129360712
**347**	0,165629	414,0725	0,125544994
**346**	0,0112298	28,0745	0,008512067
**345**	-0,0119882	-29,9705	-0,009086926
**344**	-0,0959101	-239,77525	-0,072698821

**343**	0,0758133	189,53325	0,057465663
**342**	0,0616317	154,07925	0,046716163
**341**	-0,0982898	-245,7245	-0,074502607
**340**	0,0347702	86,9255	0,026355436
**339**	0,180514	451,285	0,136827664
**338**	0,182117	455,2925	0,13804272
**337**	0,092337	230,8425	0,069990449
**336**	0,0403143	100,78575	0,030557804
**335**	0,230242	575,605	0,174520951
**334**	0,204752	511,88	0,155199806
**333**	0,0879622	219,9055	0,066674398
**332**	0,0873766	218,4415	0,06623052
**331**	0,30962	774,05	0,234688618
**330**	0,376706	941,765	0,285539082
**329**	0,370335	925,8375	0,280709933
**328**	0,19714	492,85	0,149429992
**327**	0,403825	1009,5625	0,306094991
**326**	0,817276	2043,19	0,619486387
**325**	0,31799	794,975	0,241032988
**324**	0,936611	2341,5275	0,709941028
**323**	0,966358	2415,895	0,732488933
**322**	0,766985	1917,4625	0,581366351
**321**	0,900962	2252,405	0,682919471
**320**	1,25257	3131,425	0,94943454
**319**	1,46974	3674,35	1,114047056
**318**	1,35443	3386,075	1,026643321
**317**	1,51093	3777,325	1,145268631
**316**	2,1112	5278	1,600266812
**315**	1,99186	4979,65	1,50980838
**314**	2,55771	6394,275	1,938716573
**313**	2,23978	5599,45	1,697729064
**312**	2,60288	6507,2	1,972954945
**311**	2,90215	7255,375	2,199798375
**310**	3,24893	8122,325	2,462653872
**309**	3,26255	8156,375	2,472977685
**308**	3,25726	8143,15	2,468967922
**307**	3,55347	8883,675	2,693491905
**306**	3,79064	9476,6	2,873264205
**305**	3,30769	8269,225	2,507193318
**304**	3,49303	8732,575	2,647679037
**303**	3,65719	9142,975	2,772110545
**302**	3,75378	9384,45	2,845324723
**301**	3,73557	9338,925	2,831521739
**300**	4,04728	10118,2	3,067794555
**299**	4,49757	11243,925	3,409109514
**298**	4,1346	10336,5	3,133982172
**297**	4,50355	11258,875	3,41364229
**296**	4,66739	11668,475	3,537831241

**295**	4,23045	10576,125	3,206635438
**294**	5,40447	13511,175	4,096529925
**293**	5,2372	13093	3,969741071
**292**	5,77874	14446,85	4,380222546
**291**	5,3931	13482,75	4,087911588
**290**	5,83668	14591,7	4,42414044
**289**	5,95453	14886,325	4,513469468
**288**	5,91351	14783,775	4,482376751
**287**	6,46694	16167,35	4,901870717
**286**	6,35853	15896,325	4,819697108
**285**	8,00074	20001,85	6,064474562
**284**	7,59444	18986,1	5,756503547
**283**	7,84919	19622,975	5,949601298
**282**	8,43413	21085,325	6,392979504
**281**	8,11056	20276,4	6,147716937
**280**	8,82744	22068,6	6,691104239
**279**	7,91855	19796,375	6,002175429
**278**	8,57804	21445,1	6,502061731
**277**	8,52826	21320,65	6,464329028
**276**	8,48301	21207,525	6,430030016
**275**	7,84431	19610,775	5,94590231
**274**	7,73147	19328,675	5,860370808
**273**	8,0453	20113,25	6,098250561
**272**	7,25386	18134,65	5,498347584
**271**	7,21191	18029,775	5,466549936
**270**	6,69367	16734,175	5,07372961
**269**	6,32994	15824,85	4,798026196
**268**	5,57663	13941,575	4,227025347
**267**	4,89786	12244,65	3,712525014
**266**	4,80467	12011,675	3,641888
**265**	3,64094	9102,35	2,759793221
**264**	3,24267	8106,675	2,457908859
**263**	2,48478	6211,95	1,88343642
**262**	2,15737	5393,425	1,635263174
**261**	1,1748	2937	0,890485719
**260**	0,493709	1234,2725	0,374226093
**259**	-0,595266	-1488,165	-0,451205203
**258**	-1,53945	-3848,625	-1,166886484
**257**	-3,16377	-7909,425	-2,398103511
**256**	-5,01369	-12534,225	-3,800322903
**255**	-6,05618	-15140,45	-4,590519071
**254**	-8,11801	-20295,025	-6,153363956
**253**	-9,46223	-23655,575	-7,172268207
**252**	-11,3297	-28324,25	-8,58779031
**251**	-12,177	-30442,5	-9,230034564
**250**	-13,165	-32912,5	-9,9789279
**249**	-14,835	-37087,5	-11,24476987
**248**	-15,918	-39795	-12,06567218

**247**	-16,3988	-40997	-12,4301134
**246**	-18,2236	-45559	-13,8132921
**245**	-19,1244	-47811	-14,49608878
**244**	-19,9221	-49805,25	-15,10073677
**243**	-21,4392	-53598	-16,25068219
**242**	-22,6685	-56671,25	-17,18247832
**241**	-24,0986	-60246,5	-18,26647869
**240**	-24,5171	-61292,75	-18,58369717
**239**	-24,6689	-61672,25	-18,69875993
**238**	-24,5026	-61256,5	-18,57270632
**237**	-24,0983	-60245,75	-18,26625129
**236**	-22,777	-56942,5	-17,26472015
**235**	-21,5548	-53887	-16,33830574
**234**	-19,1994	-47998,5	-14,55293797
**233**	-17,1687	-42921,75	-13,01368929
**232**	-14,1935	-35483,75	-10,7585198
**231**	-12,1725	-30431,25	-9,226623613
**230**	-10,3688	-25922	-7,859438482
**229**	-7,2589	-18147,25	-5,502167849
**228**	-4,32955	-10823,875	-3,281752168
**227**	-2,05302	-5132,55	-1,556167
**226**	0,625187	1562,9675	0,473884998
**225**	2,47556	6188,9	1,876447759
**224**	4,70458	11761,45	3,56602086
**223**	6,08915	15222,875	4,615509975
**222**	7,17644	17941,1	5,439664059
**221**	8,02516	20062,9	6,082984658
**220**	7,70398	19259,95	5,839533685
**219**	6,87114	17177,85	5,208249955
**218**	7,54663	18866,575	5,720264083
**217**	7,81175	19529,375	5,921222182
**216**	7,52711	18817,775	5,705468134
**215**	8,58697	21467,425	6,508830574
**214**	8,45725	21143,125	6,410504214
**213**	8,76681	21917,025	6,645147353
**212**	9,42808	23570,2	7,146382876
**211**	9,68364	24209,1	7,340094597
**210**	11,4029	28507,25	8,64327512
**209**	12,0003	30000,75	9,096097872
**208**	12,1007	30251,75	9,172199988
**207**	14,1605	35401,25	10,73350615
**206**	16,7203	41800,75	12,67380692
**205**	16,7735	41933,75	12,71413195
**204**	19,5621	48905,25	14,82786065
**203**	21,9164	54791	16,61239464
**202**	22,7995	56998,75	17,28177491
**201**	22,7036	56759	17,20908374
**200**	24,2078	60519,5	18,34925111

**199**	23,8393	59598,25	18,06993208
**198**	23,1784	57946	17,56897702
**197**	24,1677	60419,25	18,31885574
**196**	21,9197	54799,25	16,614896
**195**	18,1726	45431,5	13,77463465

Table 1. EDC data

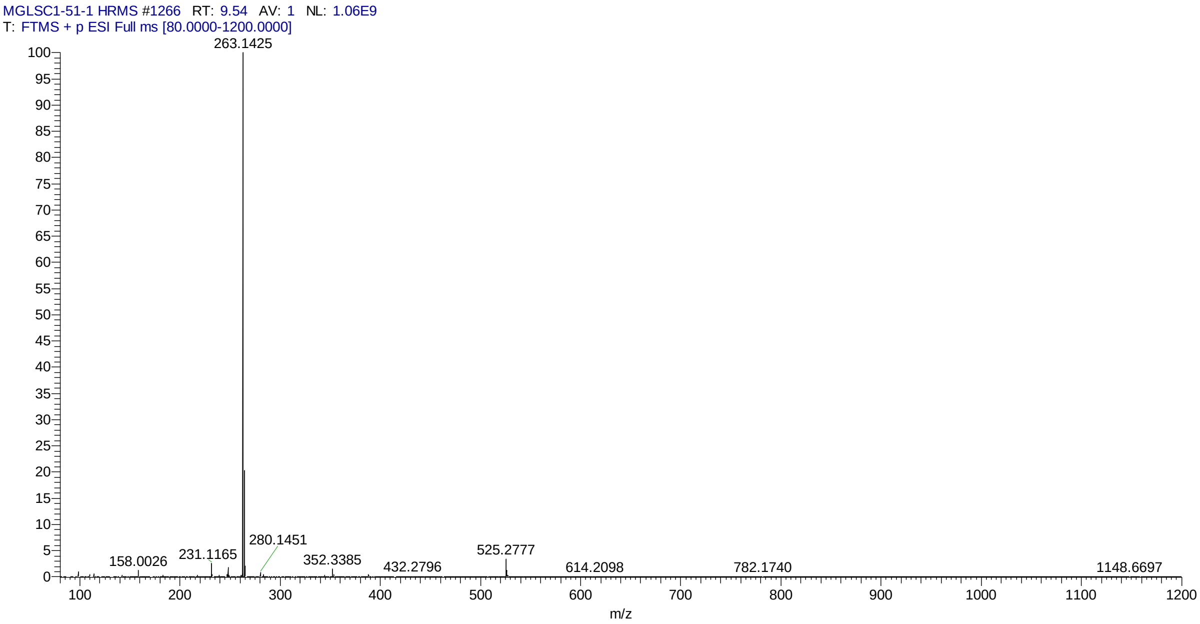

Figure S34. HRESIMS of 2-methoxy-1,7-dimethyl-5-vinyl-9,10-dihydrophenanthren-10-ol (**8**) (positive ionisation mode)

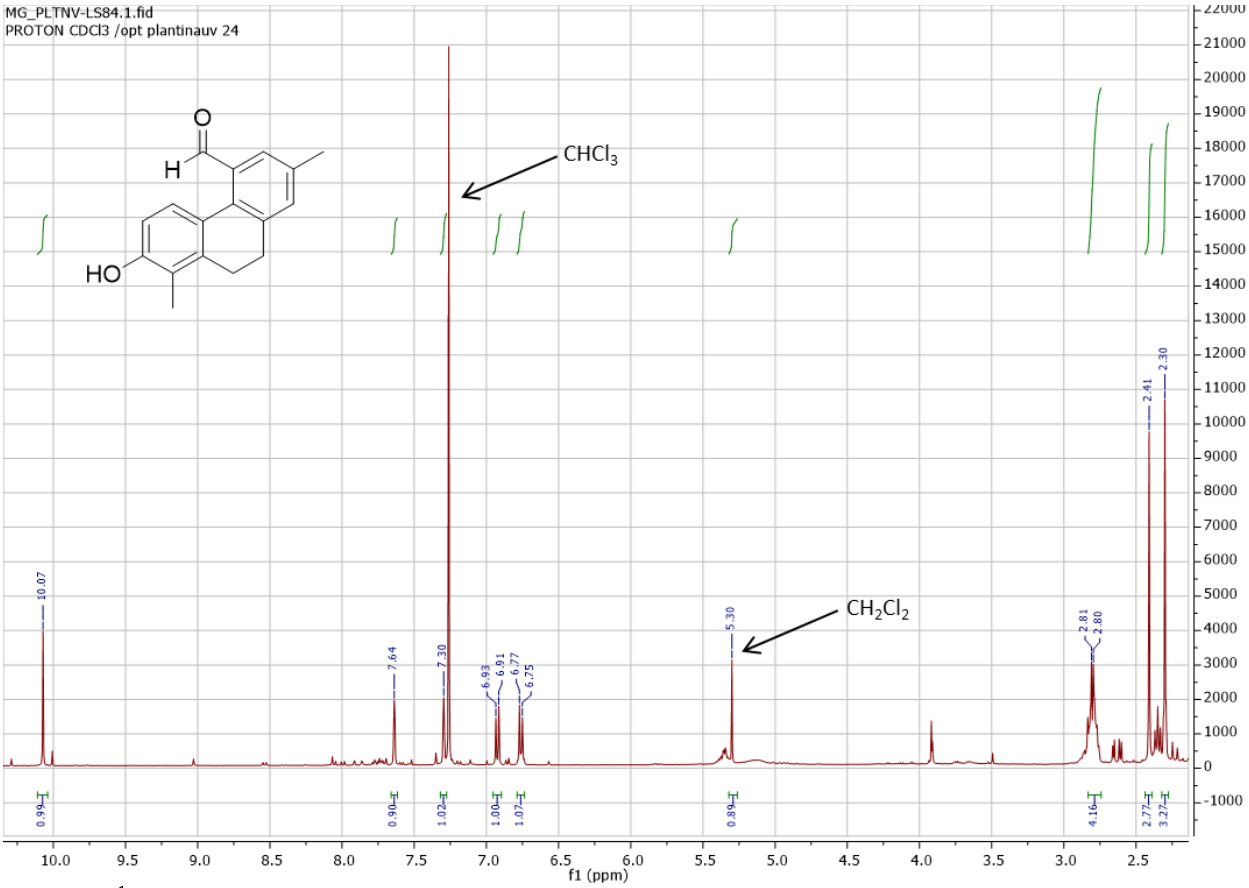

Figure S35. ^{1}H-NMR spectrum of 2-hydroxy-1,7-dimethyl-9,10-dihydrophenanthrene-5-carbaldehyde (**9**) (400MHz, $CDCl_3$)

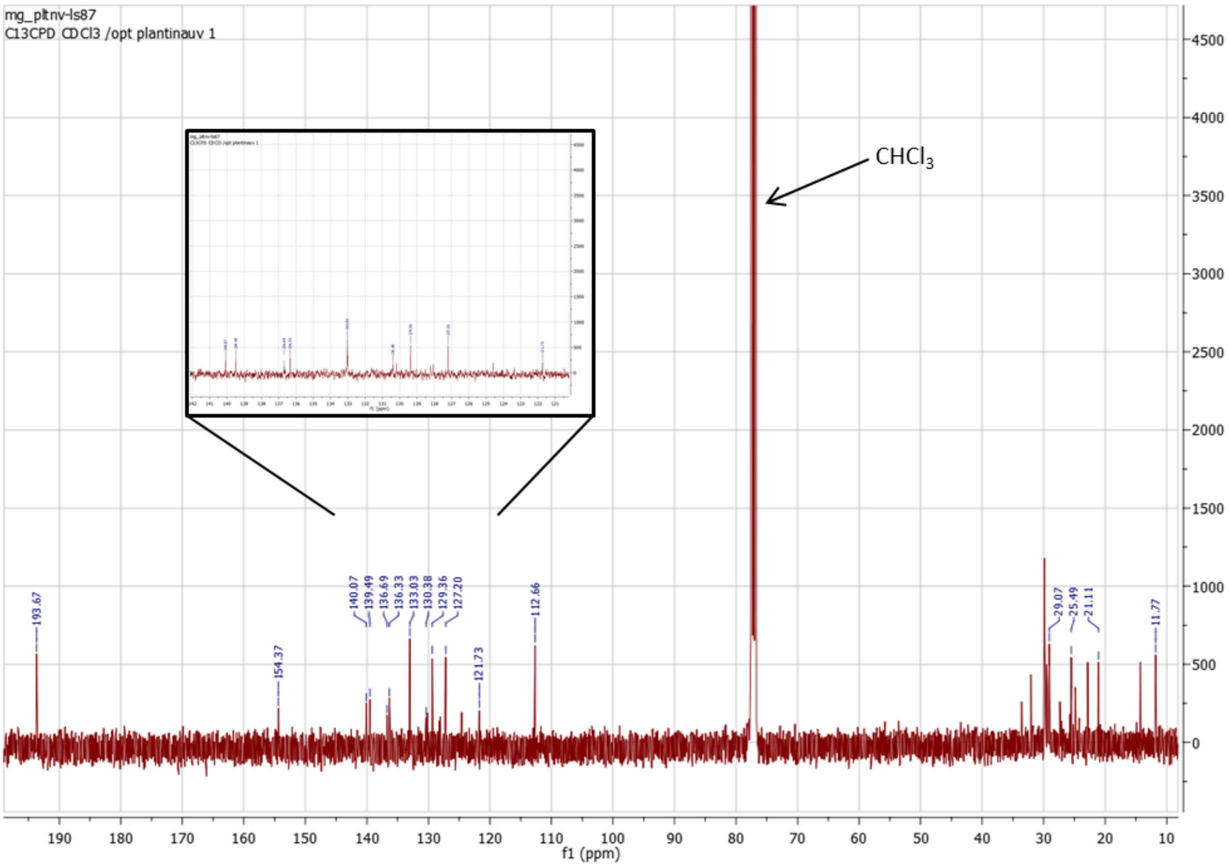

Figure S36. ^{13}C-NMR spectrum of 2-hydroxy-1,7-dimethyl-9,10-dihydrophenanthrene-5-carbaldehyde (**9**) (100MHz, $CDCl_3$)

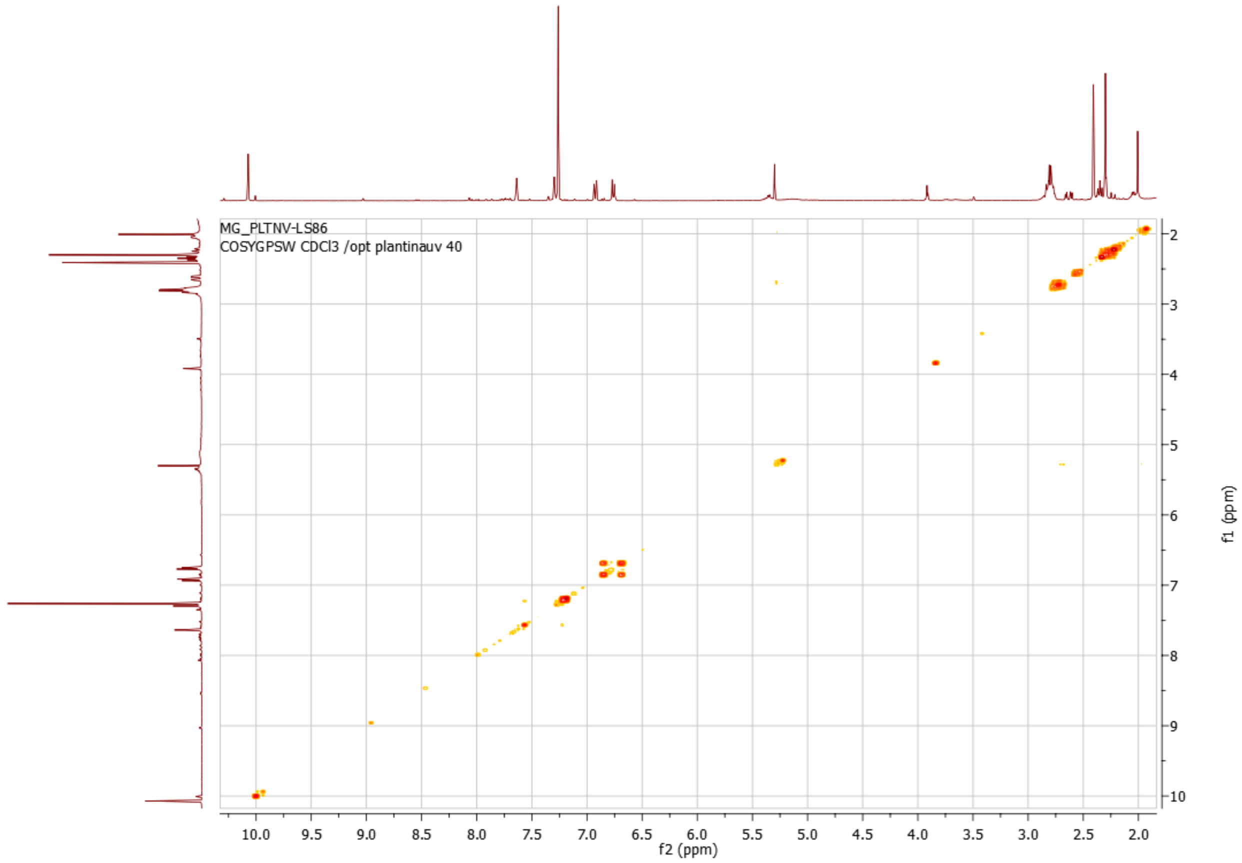

Figure S37. ^{1}H-^{1}H COSY spectrum of 2-hydroxy-1,7-dimethyl-9,10-dihydrophenanthrene-5-carbaldehyde (**9**) (400MHz, $CDCl_3$)

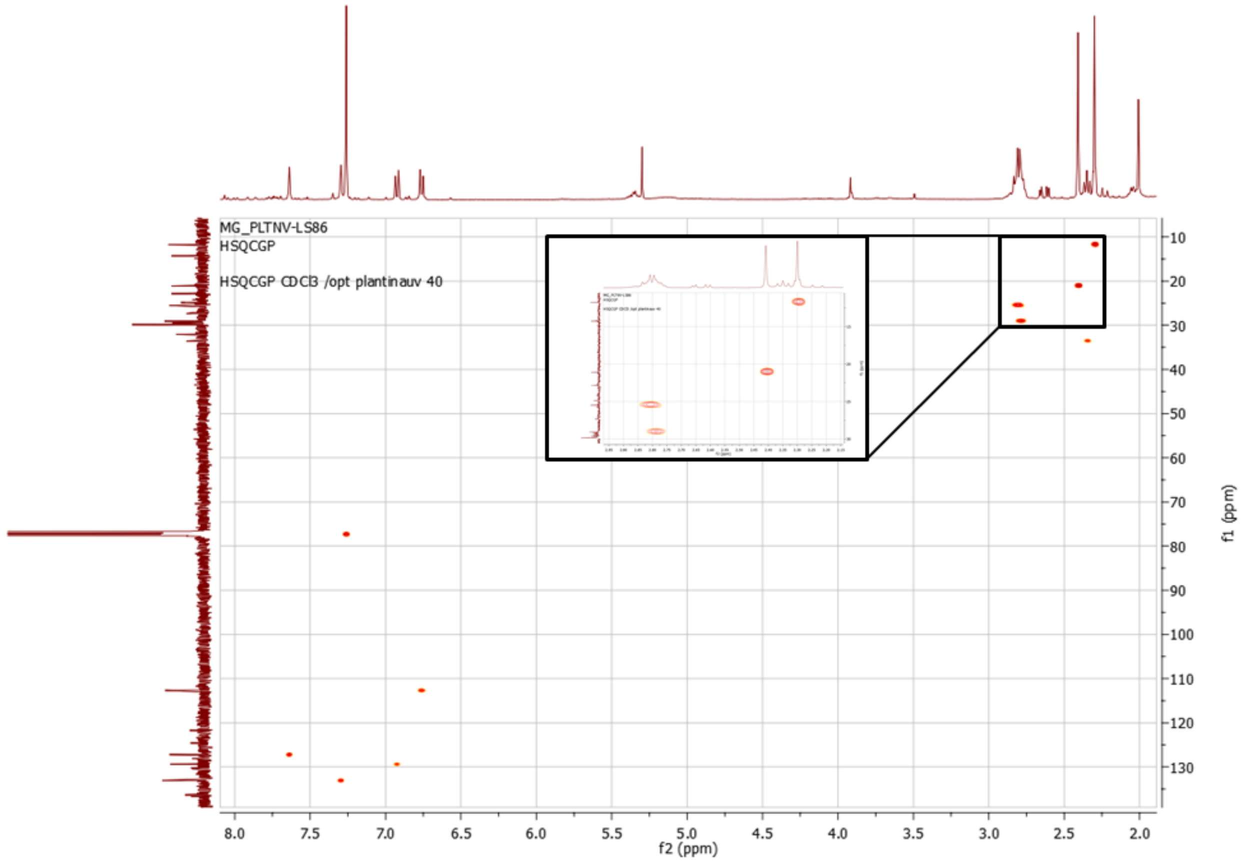

Figure S38. ^{1}H-^{13}C HSQC spectrum of 2-hydroxy-1,7-dimethyl-9,10-dihydrophenanthrene-5-carbaldehyde (**9**) (400MHz, $CDCl_3$)

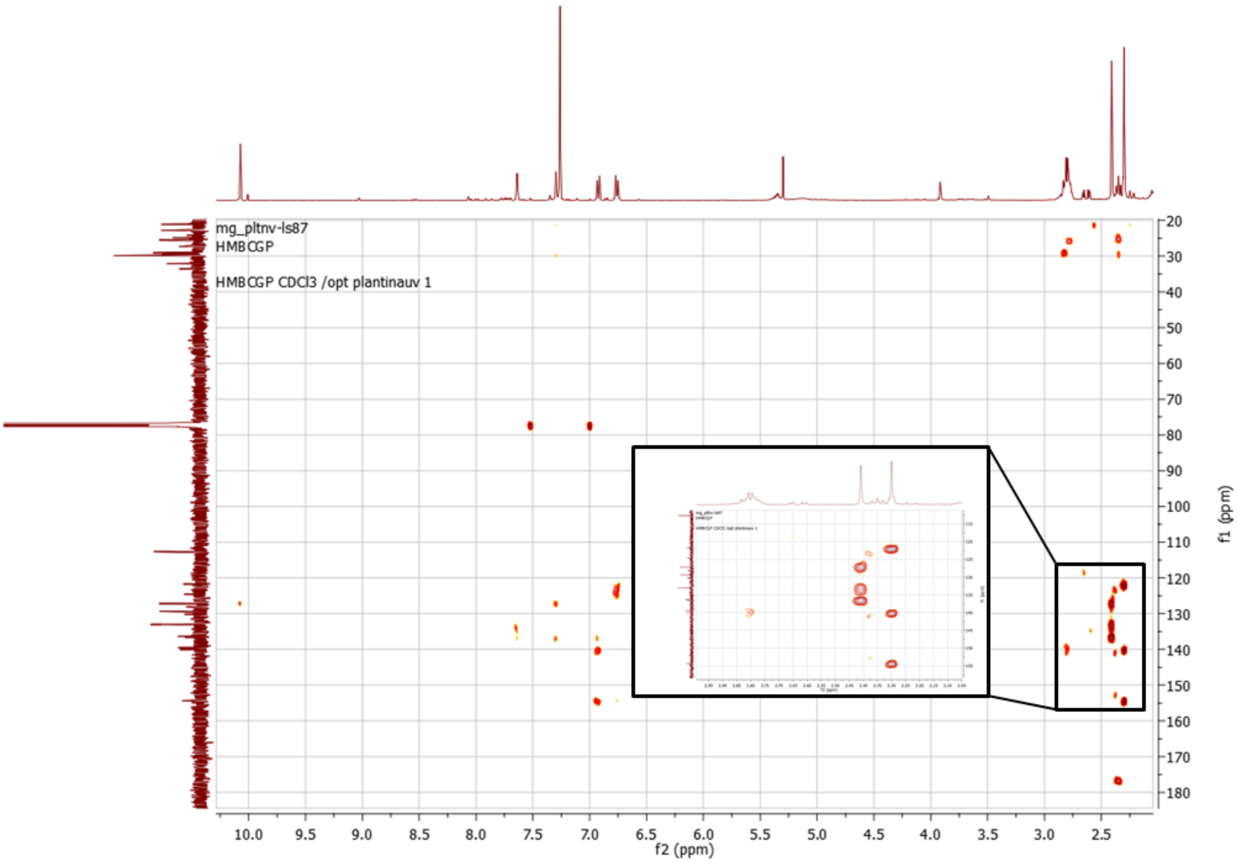

Figure S39. ^{1}H-^{13}C HMBC spectrum of 2-hydroxy-1,7-dimethyl-9,10-dihydrophenanthrene-5-carbaldehyde (**9**) (400MHz, $CDCl_3$)

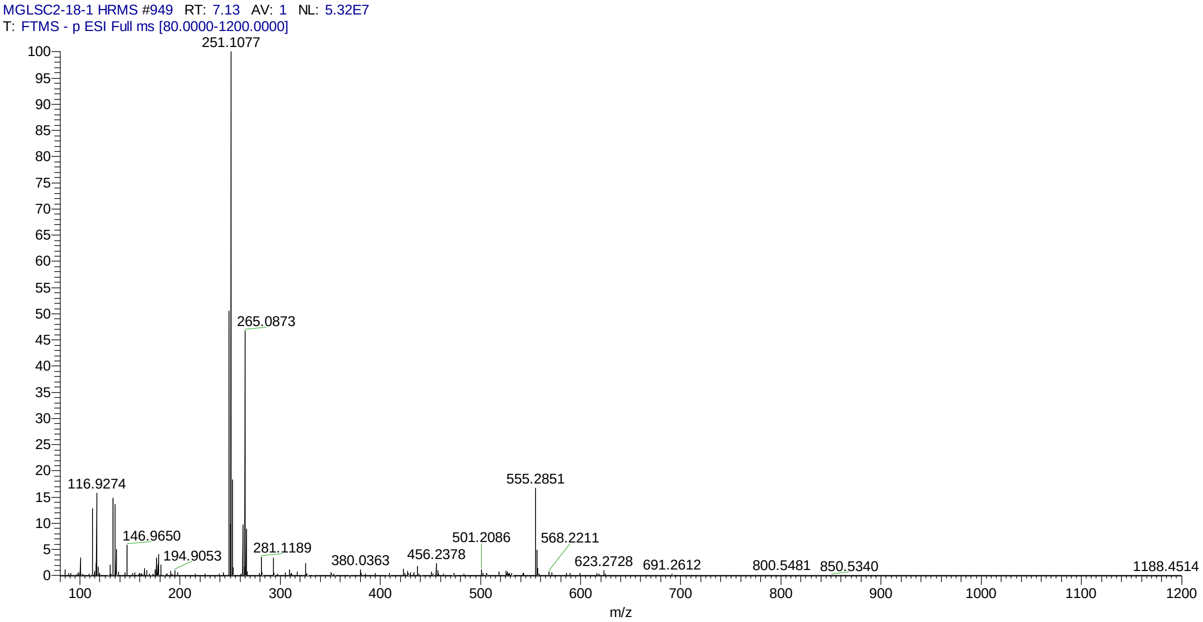

Figure S40. HRESIMS of 2-hydroxy-1,7-dimethyl-9,10-dihydrophenanthrene-5-carbaldehyde (**9**) (negative ionisation mode)
